# Longitudinal Changes in Youth Mental Health From Before to During the COVID-19 Pandemic

**DOI:** 10.1001/jamanetworkopen.2024.30198

**Published:** 2024-08-01

**Authors:** Courtney K. Blackwell, Guojing Wu, Aruna Chandran, Jessica Arizaga, Michelle Bosquet Enlow, Patricia A. Brennan, Phoebe Burton, Nicole R. Bush, David Cella, Caroline Cummins, Viren A. D’Sa, Jean A. Frazier, Jody M. Ganiban, Richard Gershon, Daphne Koinis-Mitchell, Leslie D. Leve, Christine T. Loftus, Natalia Lukankina, Amy Margolis, Sara S. Nozadi, Rosalind J. Wright, Robert O. Wright, Qi Zhao, Kaja Z. LeWinn, the Environmental influences on Child Health Outcomes Program Collaborators

**Affiliations:** Department of Medical Social Sciences, Northwestern University Feinberg School of Medicine, Chicago, Illinois; Department of Epidemiology, Johns Hopkins Bloomberg School of Public Health, Baltimore, Maryland; Department of Epidemiology, Johns Hopkins Bloomberg School of Public Health, Baltimore, Maryland; Department of Psychiatry and Behavioral Sciences, University of California School of Medicine, San Francisco; Department of Psychiatry and Behavioral Sciences, Boston Children’s Hospital, Boston, Massachusetts; Department of Psychiatry, Harvard Medical School, Boston, Massachusetts; Department of Psychology, Emory University, Atlanta, Georgia; Department of Pediatrics, Rhode Island Hospital, Providence; Department of Pediatrics, The Warren Alpert Medical School of Brown University, Providence, Rhode Island; Department of Psychiatry and Behavioral Sciences, University of California School of Medicine, San Francisco; Department of Pediatrics, University of California School of Medicine, San Francisco; Department of Medical Social Sciences, Northwestern University Feinberg School of Medicine, Chicago, Illinois; Department of Pediatrics, Rhode Island Hospital, Providence; Department of Pediatrics, The Warren Alpert Medical School of Brown University, Providence, Rhode Island; Department of Pediatrics, Rhode Island Hospital, Providence; Department of Pediatrics, The Warren Alpert Medical School of Brown University, Providence, Rhode Island; Hasbro Children’s Hospital, Providence, Rhode Island; Eunice Kennedy Shriver Center, UMass Chan Medical School, Worcester, Massachusetts; Department of Psychiatry, UMass Chan Medical School, Worcester, Massachusetts; Department of Pediatrics, UMass Chan Medical School, Worcester, Massachusetts; Department of Clinical/Developmental Psychology, George Washington University, Washington, DC; Department of Medical Social Sciences, Northwestern University Feinberg School of Medicine, Chicago, Illinois; Department of Pediatrics, Rhode Island Hospital, Providence; Department of Pediatrics, The Warren Alpert Medical School of Brown University, Providence, Rhode Island; College of Education, University of Oregon, Eugene; Department of Environmental and Occupational Health Sciences, School of Public Health, University of Washington, Seattle; Department of Pediatrics, Rhode Island Hospital, Providence; Department of Pediatrics, The Warren Alpert Medical School of Brown University, Providence, Rhode Island; Hasbro Children’s Hospital, Providence, Rhode Island; Department of Psychiatry, Columbia University Irving Medical Center, New York, New York; Community Environmental Health Program, University of New Mexico Health Sciences Center, Albuquerque; Department of Pediatrics, Icahn School of Medicine at Mount Sinai, New York, New York; Department of Pediatrics, Icahn School of Medicine at Mount Sinai, New York, New York; Department of Environmental Medicine & Public Health, Icahn School of Medicine at Mount Sinai, New York, New York; Department of Preventive Medicine, University of Tennessee Health Science Center, Memphis; Department of Psychiatry and Behavioral Sciences, University of California School of Medicine, San Francisco

## Abstract

**IMPORTANCE:**

Robust longitudinal studies of within-child changes in mental health associated with the COVID-19 pandemic are lacking, as are studies examining sources of heterogeneity in such changes.

**OBJECTIVE:**

To investigate within-child changes, overall and between subgroups, in youth mental health from prepandemic to midpandemic.

**DESIGN, SETTING, AND PARTICIPANTS:**

This cohort study used longitudinal prepandemic and midpandemic data from the Environmental influences on Child Health Outcomes (ECHO) Program, collected between January 1, 2015, and March 12, 2020 (prepandemic), and between March 13, 2020, and August 31, 2022 (midpandemic). Data were analyzed between December 1, 2022, and June 1, 2024. The sample included 9 US-based observational longitudinal pediatric ECHO cohorts. Cohorts were included if they collected the Child Behavior Checklist (CBCL) School Age version before and during the pandemic on more than 20 participants of normal birth weight aged 6 to 17 years.

**EXPOSURE:**

The COVID-19 pandemic.

**MAIN OUTCOMES AND MEASURES:**

Prepandemic to midpandemic changes in CBCL internalizing, externalizing, depression, anxiety, and attention-deficit/hyperactivity disorder (ADHD) scores were estimated, and differences in outcome trajectories by child sociodemographic characteristics (age, sex, race, ethnicity, and poverty level) and prepandemic mental health problems were examined using established CBCL clinical score thresholds.

**RESULTS:**

A total of 1229 participants (mean [SD] age during the pandemic, 10.68 [2.29] years; 625 girls [50.9%]) were included. The sample was socioeconomically diverse (197 of 1056 children [18.7%] lived at ≤130% of the Federal Poverty Level; 635 (51.7%) identified as White, 388 (31.6%) as Black, 147 (12.0%) as multiracial, 40 (3.3%) as another race, and 118 (9.6%) as Hispanic). Generalized linear mixed-effects models revealed minor decreases in externalizing problems (β = −0.88; 95% CI, −1.16 to −0.60), anxiety (β = −0.18; 95% CI, −0.31 to −0.05), and ADHD (β = −0.36; 95% CI, −0.50 to −0.22), but a minor increase in depression (β = 0.22; 95% CI, 0.10 to 0.35). Youth with borderline or clinically meaningful prepandemic scores experienced decreases across all outcomes, particularly externalizing problems (borderline, β = −2.85; 95% CI, −3.92 to −1.78; clinical, β = −4.88; 95% CI, −5.84 to −3.92). Low-income (β = −0.76; 95% CI, −1.14 to −0.37) and Black (β = −0.52; 95% CI, −0.83 to −0.20) youth experienced small decreases in ADHD compared with higher income and White youth, respectively.

**CONCLUSIONS AND RELEVANCE:**

In this longitudinal cohort study of economically and racially diverse US youth, there was evidence of differential susceptibility and resilience for mental health problems during the pandemic that was associated with prepandemic mental health and sociodemographic characteristics.

## Introduction

Nearly 50% of youth have a mental health disorder in their lifetime.^1^ The narrative emphasized by recent cross-sectional studies^2–6^ is that the COVID-19 pandemic and related containment strategies exacerbated mental health risks. The few longitudinal studies examining within-child change using the same prepandemic and midpandemic mental health measures focused only on the first year of the pandemic and either took place outside the US^7–19^ or primarily used data from the US-based Adolescent Brain Cognitive Development study, which included children aged 14 to 16 years.^20–22^ These studies suggest an initial increase in mental health problems in the early phases of the pandemic^7–9,12–15,20,21^ followed by recovery (although not necessarily to prepandemic levels^10,11,14,16,21,22^), and, overall, clinically small but statistically significant increases in mental health problems, particularly depression.

Despite theoretical support for the notion that the pandemic affected subgroups of children differently,^23–25^ few longitudinal studies have investigated such heterogeneity.^17–19^ Work emphasizing that female individuals fared worse than male individuals has been conducted primarily in older samples,^7–9,17^ and research suggests the pandemic was especially stressful for adolescents^18,19^ but within-study age-based comparisons are limited, as is research on pandemic-related mental health changes in younger populations. For other subgroups with vulnerability to pandemic-related social disruptions, such as prepandemic mental health problems,^26^ lower family income,^27,28^ and minoritized race and ethnicity,^27,28^ smaller studies were unable to detect subgroup differences, and results from larger studies were inconsistent.^18,19^ A more nuanced understanding of the pandemic’s impact on youth mental health is, therefore, critically needed.

This study addresses prior limitations by leveraging individual-level data from before and during the pandemic for 6- to 17-year-olds participating in the National Institutes of Health–funded Environmental influences on Child Health Outcomes (ECHO) Program.^29^ We hypothesized the following: first, the pandemic was associated with increases in youth mental health problems (hypothesis 1 [H1]), and second, greater increases in mental health problems were associated with having prepandemic mental health problems, being female, aged 12 years or older, lower family income, or being in a minoritized racial or ethnic group (hypothesis 2 [H2]).

## Methods

### Participants

For this cohort study, children were included if they had a Child Behavior Checklist (CBCL) School Age version (aged 6–18 years) assessment before the pandemic (from January 1, 2015, to March 12, 2020) and during the pandemic (March 13, 2020, to August 31, 2022), and were aged 6 to 17 years before the pandemic, of normal birth weight, and from an ECHO cohort with more than 20 participants contributing data. Local institutional review boards (IRBs) and/or the central ECHO IRB (Western IRB) reviewed all methods and procedures. Written informed consent or the parents’ or guardians’ permission was obtained, along with child assent as appropriate. This study followed the Strengthening the Reporting of Observational Studies in Epidemiology (STROBE) reporting guidelines for cohort studies.^30^

Mental health was measured using the CBCL, a parent-report measure of youth behavior and mental health.^31,32^ We examined the internalizing and externalizing problems composites, as well as *Diagnostic and Statistical Manual of Mental Disorders* (Fifth Edition)–oriented scales for depression, anxiety, and attention-deficit/hyperactivity disorder (ADHD). These 5 outcomes are the most common pediatric mental health conditions in the US,^33^ were increasing in prevalence even before the pandemic,^34,35^ and were hypothesized as being most impacted by the pandemic.^36^ In line with recommendations for examining within-person change,^37^ analyses used raw CBCL scores. If children had multiple assessments during the pandemic, the earliest (closest to March 13, 2020) was selected. Child race and ethnicity were reported by the caregiver in accordance with National Institutes of Health requirements related to reporting of individual-level participant data.

### Statistical Analysis

We used generalized linear mixed-effects models^38^ to estimate mental health changes from before to during the pandemic (ie, midpandemic). For succinctness, we use the term *midpandemic* to describe the second assessment but acknowledge that children were assessed throughout the pandemic. Models included 2 repeated measures of child mental health as the dependent variable. Visit occasion (prepandemic or midpandemic) was the primary independent variable, with the prepandemic measurement as the reference time point. The visit coefficient estimated change in CBCL scores between prepandemic and midpandemic (H1). Models included random intercepts both for child and cohort to allow prepandemic raw scores to vary between children and between cohorts, respectively. Models were also adjusted for covariates identified a priori. Sociodemographic covariates included child midpandemic age (6–11 years vs 12–17 years, based on the CBCL age-based norming categories), sex at birth, caregiver-identified child race (owing to sample size limitations, categories were collapsed to Black, White, and other race, which includes American Indian or Alaska Native, Asian Indian, Other Asian [Chinese, Filipino, Japanese, Korean, Vietnamese, Other Asian, and do not know], Native Hawaiian, Other Pacific Islander, some other race, prefer not to answer, and do not know) and ethnicity (Hispanic or non-Hispanic), caregiver highest educational attainment (less than high school, high school, some college, or bachelor’s degree or higher), and family income adjusted for household size and then categorized as percentage of the Federal Poverty Level (FPL) using thresholds established in prior studies (≤130%, >130% to 350%, and >350%).^39^ We used income data available from January 1, 2017, to August 31, 2022, and selected the record closest to March 2020.

Caregiver covariates included mean-centered, self-reported depressive symptoms and perceived stress measured at least once from January 1, 2017, to August 31, 2022. Cohorts used different validated depression measures (ie, PROMIS version 1.0 Depressive Symptoms, Patient Health Questionnaire–9, Brief Symptom Inventory, Short Form–36 Mental Health, and Center for Epidemiological Studies Depression Scale), which were harmonized to the standard PROMIS T score metric (mean [SD], 50 [10]) using the PROsetta Stone score-linking method.^40–46^ Caregiver-perceived stress was measured using the Perceived Stress Scale^47,48^ and scored on the T score metric. For caregivers with multiple assessments, scores were averaged.

Models were also adjusted for number of months between March 13, 2020, and the midpandemic CBCL assessment. We used the multiple imputation by chained equations method (25 imputations, each with 10 iterations) to impute missing covariate data.^49^

Building on H1 models, we included an interaction term between visit occasion and each hypothesized variable associated with mental health change (H2): prepandemic mental health, sex, age, income, race, and ethnicity. We defined prepandemic mental health problems using the nationally normed and clinically validated CBCL cutoff scores indicating normal (T score <65), borderline (T score = 65–70), and clinically meaningful (T score >70) behavior problems^31,32^; because this score was not in H1 models, models testing the interaction between visit and prepandemic mental health also included the main effect estimate. We conceptualized race and ethnicity as social constructs that reflect membership in marginalized groups that have experienced inequitable burdens of COVID-19 infection and disease severity^27,28^ and fewer returns on educational investments with respect to health and financial gains^28,50^ that may buffer pandemic-related experiences. We examined both visit-by-race category and visit-by-ethnicity interactions using the largest subgroup as the reference (White and non-Hispanic, respectively). In post hoc exploratory analyses responsive to our findings, we tested the 3-way interactions of prepandemic mental health by age by visit and prepandemic mental health by sex by visit to examine associations within subgroups of children for whom intersectionality (ie, the intersections between individual identities that manifest in specific positions within societal structures and developmental contexts)^51^ may have exacerbated or mitigated the impact of the pandemic on mental health.

Statistical significance was evaluated using a likelihood ratio test; if the interaction term was significant at *P* < .05 for at least 1 outcome, least squares (LS) prepandemic and midpandemic means, as well as their differences, were estimated within groups to facilitate interpretation of interaction terms. We further contextualize effect sizes using Cohen *d* (small, 0.2 SD; medium, 0.5 SD; and large, 0.8 SD^52^) according to the SD of each outcome at baseline. Given that the average pandemic impact on youth is estimated to be small (approximately 0.3 SD), we focus interpretations on effects that are small or greater.^17,53,54^ Data were analyzed between December 1, 2022, and June 1, 2024, using R statistical software version 4.1.0 (R Project for Statistical Computing).

We were interested in whether the pandemic had a greater impact on the mental health of adolescents (aged 12 years or older) as indicated by prior research, and, thus, included a dichotomous indicator of age as a covariate in all our models to facilitate interpretation across models. In sensitivity analyses, we adjusted for continuous child age in main models to examine whether this altered effect estimates. In addition, we examined whether adjustment for time between the prepandemic to midpandemic assessments impacted effect estimates.

## Results

The analytic sample included 1229 participants (625 girls [50.9%]) from 9 ECHO cohorts (eFigure 1 and eTable 1 in Supplement 1). Children were a mean (SD) of 8.07 (1.83) years old before the pandemic and 10.68 (2.29) years old midpandemic (eFigures 2 and 3 in Supplement 1). The analytic sample largely reflected the characteristics of the eligible cohort sample (5319 individuals) for child age, sex, race, ethnicity, and family income (Table 1); the analytic sample had somewhat higher levels of caregiver education (490 of 1180 [41.5%] vs 1350 of 4143 [32.6%] with a master’s degree or higher). Children were racially, ethnically, and economically diverse: 635 (51.7%) identified as White, 388 (31.6%) as Black, 147 (12.0%) as multiracial, 40 (3.3%) as another race, and 118 (9.6%) as Hispanic; 197 of 1056 children (18.7%) came from households at or below 130% of the FPL. Midpandemic assessments were conducted a mean (SD) of 12.52 (6.45) months (range, 0.03–29.60 months) after March 12, 2020 (see eFigures 4, 5, and 6 in Supplement 1 for assessment timing). Mean (SD) CBCL scores before the pandemic were 5.0 (5.2) for internalizing scores (median [IQR], 3 [1–7]), 5.8 (6.1) for externalizing scores (median [IQR], 4 [1–8]), 1.3 (1.8) for depression scores (median [IQR], 1 [0–2]), 2.2 (2.5) for anxiety scores (median [IQR], 1 [0–3]), and 3.4 (3.1) for ADHD scores (median [IQR], 3 [1–5]). These SDs are used to identify small, medium, and large effect sizes based on Cohen *d*.^52^ Between 2% and 8% of children entered the pandemic with borderline or clinically meaningful CBCL scores (eTable 2 in Supplement 1).

### Mean Change in Mental Health

Overall, youth experienced minor decreases in externalizing problems (β = −0.88; 95% CI, −1.16 to −0.60), anxiety (β = −0.18; 95% CI, −0.31 to −0.05), and ADHD (β = −0.36; 95% CI, −0.50 to −0.22), and a minor increase in depression (β = 0.22; 95% CI, 0.10 to 0.35) (Table 2). All effect estimates represented changes in scores that were less than 0.2 SD.

### Heterogeneous Associations

For these models, estimated subgroup effect estimates (ie, slopes capturing change in CBCL scores) are in comparison to the reference group. Therefore, in terms of absolute change over time, a significant, negative slope for a subgroup could mean that (1) all groups are declining over time but the subgroup of interest is experiencing greater declines, (2) the reference group is not changing but the subgroup of interest is declining, or (3) the reference group is increasing while the subgroup of interest is not changing (or declining). Thus, a negative slope does not necessarily equate to improvements in mental health problems. To facilitate interpretation, we also provide the LS means to estimate absolute change within subgroups.

#### Differences in Changes in Mental Health by Sociodemographic Characteristics

We did not observe differences in mental health changes by ethnicity (eTable 3 in Supplement 1). Compared with male individuals, female individuals experienced minor increases in externalizing scores (β = 0.56; 95% CI, 0.01 to 1.11) (eTable 4 in Supplement 1). Compared with older children, slopes were negative among younger children (<12 years old) for internalizing problems (β = −0.75; 95% CI, −1.40 to −0.09) and depression (β = −0.47; 95% CI, −0.76 to −0.17) (eTable 5 in Supplement 1; LS means are shown in eTable 6 in Supplement 1). With respect to income, the slope associated with change in mental health for children in families with income less than 130% FPL was negative for internalizing (β = −0.80; 95% CI, −1.56 to −0.03) and externalizing (β = −0.91; 95% CI, −1.67 to −0.15) problems, depression (β = −0.45; 95% CI, −0.79 to −0.10), and ADHD (β = −0.76; 95% CI, −1.14 to −0.37) (Table 3; see eTable 7 in Supplement 1 for LS means). Black children had slopes that were negative compared with White children for internalizing problems (β = −0.87; 95% CI, −1.50 to −0.25), depression (β = −0.48; 95% CI, −0.76 to −0.20), and ADHD (β = −0.52; 95% CI, −0.83 to −0.20) (Table 4; see eTable 8 in Supplement 1 for LS means). All effect sizes associated with broadband internalizing or externalizing scores in relation to sociodemographic characteristics were less than 0.15 SD; however, subscale score differences were relatively larger (all effect estimates were approximately 0.3–0.4 SD).

#### Differences in Changes in Mental Health by Prepandemic Mental Health

For all outcomes, children entering the pandemic with borderline or clinically meaningful CBCL scores experienced large decreases in scores compared with those with prepandemic scores in the normal range. Most notably, children experienced medium to large (changes of 0.5 to 1 SD) decreases in externalizing problems (β = −4.88; 95% CI, −5.84 to −3.92) and ADHD symptoms (β = −1.40; 95% CI, −1.91 to −0.89) (Table 5) from prepandemic to midpandemic (see LS means in eTable 9 in Supplement 1). A similar pattern of medium effect sizes was observed among children with CBCL scores in the borderline range (externalizing problems, β = −2.85; 95% CI, −3.92 to −1.78; ADHD, β = −1.28; 95% CI, −1.85 to −0.72). Children entering the pandemic with clinically meaningful internalizing problems, depression, and anxiety also experienced declines (medium effect sizes) in symptoms (internalizing problems, β = −2.87; 95% CI, −3.88 to −1.87; depression, β = −0.81; 95% CI, −1.26 to −0.35; and anxiety, β = −1.16; 95% CI, −1.63 to −0.70). For post hoc exploratory analyses, we report results for internalizing and externalizing broadband scores only given sample size limitations (see eTable 10 in Supplement 1 for 3-way interaction sample sizes). For those entering the pandemic with clinically meaningful internalizing scores, the slope for younger children (<12 years old) was negative compared with older children (β = −3.22; 95% CI, −5.58 to −0.86; medium to large effect size) (eTable 11 in Supplement 1); younger children experienced a notable reduction in symptoms (3.4 points) whereas older children increased (0.5 points) on average (eTable 12 in Supplement 1). For youth entering the pandemic with internalizing problems, we also observed differences by sex. For those with borderline scores, female individuals had a positive slope compared with male individuals (β = 3.35; 95% CI, 1.09 to 5.96; a medium to large effect size) (eTable 13 in Supplement 1); on average, male individuals experienced a 2-point decline, and female individuals experienced a 1.6-point increase (eTable 14 in Supplement 1). For female individuals entering the pandemic with clinically meaningful internalizing scores, the slope was negative compared with male individuals (β = 2.78; 95% CI, 1.09 to 5.61; medium effect size) such that female individuals experienced a 4-point decline where male individuals experienced 1.4-point decline (eTable 14 in Supplement 1). We observed no significant differences by age or sex for externalizing problems among those entering the pandemic with borderline or clinical scores. In sensitivity analyses, adjustment for age modeled continuously (eTable 15 in Supplement 1) and time between the prepandemic and midpandemic assessments (eTable 16 in Supplement 1) did not impact our findings.

## Discussion

Contrary to our hypotheses and prior cross-sectional studies, in this cohort study we found no association of the COVID-19 pandemic with average changes in internalizing problems and observed very small decreases in externalizing problems. Slight increases in depression reported here are consistent with findings from several longitudinal studies and meta-analyses,^17,20^ and collectively, this work converges on statistically significant but likely not clinically meaningful increases in youth depression, on average, associated with the pandemic. Our finding of a lack of a meaningful impact of the pandemic on child mental health in our full study sample is an important contribution, given that our within-child analysis accounts for stable characteristics of the child (eg, genetic predisposition, stable aspects of the family environment, and prior mental health–related exposures such as prior adverse childhood experiences). Prior longitudinal studies that only include outcomes measured during the pandemic (eg, Xiao et al^21^ and Ravens-Sieberer et al^16^) are more vulnerable to residual confounding by factors associated with poor mental health and are either unmeasured or not included as covariates, greatly limiting internal validity and likely overestimating the average impact of the pandemic on mental health. Although addressing youth mental health is warranted given the increasing prepandemic rates, such efforts may be better focused on subgroups. As shown here, although average effect sizes were small, some subgroups of youth experienced moderate changes.

We found that children entering the pandemic with clinically meaningful mental health problems experienced notable improvements in their mental health, suggesting that average associations of the pandemic with mental health tell only part of the story. Youth entering the pandemic with borderline or clinical range CBCL scores experienced medium-to-large decreases in all scores (approximately 0.5–1.0 SDs), particularly for externalizing problems, suggesting real and meaningful reductions in mental health problems for these children.

Limited work has examined mental health changes for youth with preexisting mental health problems, but those that do suggest similar results as those found here. Several studies with older children and adolescents found symptom improvement during the early months of the pandemic for youth who entered with preexisting mental health problems.^15,20,22,55^ We extend this work across a larger age range and beyond the first year of the pandemic, suggesting that these unexpected decreases in mental health problems may persist. One potential explanation is that pandemic-related social restrictions (eg, school closures) represented a break from stressful social environments that may have been more impactful for youth with preexisting mental health problems.^2,56–59^ Academic stress and school-related pressures, as well as negative social interactions such as bullying, social comparison, and academic and extracurricular competition, can contribute to psychological distress^60–62^; for youth lacking the necessary coping skills and resources, such as those with mental health problems, these school and social stressors can have detrimental psychological impacts. Indeed, seasonal patterns in psychiatric hospital admissions and diagnoses, suicidality, and depression track with the school year but only in youth, not adults, suggesting school-related stressors contribute to poor youth mental health.^59^ Thus, a pause during COVID-19 from such stressful contexts may have improved mental health for these youth. The current study cannot draw finite conclusions as to whether school closures or social restrictions in particular were associated with improvements in youth mental health, and additional investigations into this potential pathway to improved outcomes for these vulnerable youth is warranted to clarify these relationships.^36,63^ Such work can help inform parents, practitioners, and policymakers on potential adaptive choices and strategies to combat the current youth mental health crisis.

Although exploratory analyses should be interpreted with caution, we found preliminary evidence to suggest that decreases in internalizing scores among those who entered the pandemic in the clinically meaningful range were more notable in younger children. With respect to sex, female individuals with borderline internalizing symptoms experienced increases in symptoms during the pandemic, whereas male individuals experienced decreases. In contrast, female individuals with clinically meaningful internalizing problems experienced the largest decreases in symptoms. It is worth noting that we did not observe clinically meaningful main effect estimates of either age or sex on pandemic-related changes in mental health, but this preliminary evidence suggests that both characteristics, in combination with preexisting mental health problems, may have influenced how youth experienced the pandemic. Future work with larger, clinically enriched samples is needed to further investigate heterogeneous effects of the pandemic on youth mental health.

We also found that other groups of children who might be considered more vulnerable to mental health problems in the context of pandemic-related disruptions generally fared better compared with their peers. Low-income and Black youth experienced decreases (0.3–0.4 SD) in ADHD symptoms, whereas higher income and White youth largely stayed the same. In contrast, high-income and White youth experienced increases in depression symptoms, whereas low-income and Black youth largely stayed the same. Although low-income and Black families experienced disproportionately larger financial hardships, COVID-19 infection rates, and reduced health care access,^27^ our results suggest the pandemic had null or small positive outcomes for these youth. One possible reason for such findings is that schools in lower-income communities are more likely to be underresourced, overcrowded,^64^ and have more school-based violence^65,66^; furthermore, schools can be threatening spaces for Black youth.^67,68^ Thus, observed improvements in mental health, although small, may be related to reduced exposure to these perpetually stressful environments during school closures and remote schooling. However, these youth may also have experienced substantial academic setbacks due to economic and racial and ethnic digital divides and lacking the technology infrastructure necessary to participate in remote learning.^69^ School closures also resulted in limited access to school-based services such as free or reduced price lunch, which are disproportionately used by lower income and minoritized youth.^69^ Understanding subgroup-specific impacts of school closures and other pandemic-related social restrictions on a broad range of neurodevelopmental and academic outcomes is essential for accurately capturing the pandemic’s impact on youth.

Although these sociodemographic differences were small according to Cohen *d* criteria, these estimates were observed using a robust, within-child design. Burgeoning evidence suggests application of less-robust study designs in child mental health research has overestimated well-studied relationships. For example, a recent meta-analysis^70^ examining the association between childhood maltreatment and child mental health estimated in robust, quasi-experimental studies (including within-child study designs) concluded that these associations were smaller (Cohen *d* = 0.31, small effect size) compared with estimates from less-robust designs (Cohen *d* = 0.56, medium effect size). Thus, effect estimates observed here can be considered comparable in magnitude to the impact of childhood maltreatment on child mental health. Finally, such differences are important to interrogate in future research as they may both point to children who are particularly vulnerable to depression during the pandemic as well as to potential pathways by which to reduce inequalities in ADHD outcomes.

### Limitations

This study has limitations that should be mentioned. The ECHO Cohort is not nationally representative, and our analytic sample had a smaller proportion of Hispanic youth and families with lower education compared with our eligible sample, similar to other studies that continued during the pandemic.^71^ Thus, our results may not generalize to the most disadvantaged youth. Second, our outcome data are parent-reported, and parents may underreport youth mental health symptoms.^72^ However, parent report enabled reliable and valid measurement across a large age range, and cross-informant CBCL analyses suggest moderate correlations between parent and youth report that are larger than other instruments.^32^ The CBCL also has high test-retest reliability and scale score stability,^32^ suggesting that parent informants are internally consistent across time and, therefore, the absolute change in mental health problems is reliably estimated, even if individual time points are subject to potential underestimation. In addition, because our analysis includes only 2 measurement occasions, we are unable to completely address concerns that our findings, particularly those focused on children with prepandemic mental health problems, are due to regression to the mean. However, we do find evidence that scores in subgroups of these at-risk children increase and decrease (eg, male individuals vs female individuals with borderline internalizing symptoms prepandemic) in a manner supported by prior evidence. This increases our confidence that these results are not a statistical artifact.

## Conclusions

Overall, our results suggest the pandemic had a minimal impact on child mental health on average. Importantly, however, changes in mental health—some positive, some negative—depended on individual characteristics, such as prepandemic mental health and sociodemographic characteristics. These differences are critical for understanding how best to support different youth during social disruptions and future related research may yield novel insights into causes of youth mental health problems.

## Supplementary Material

supplement **eFigure 1**. Participant flow diagram
**eFigure 2**. Distribution of child ages at the pre-pandemic CBCL assessment
**eFigure 3**. Distribution of child ages at the mid-pandemic CBCL assessment
**eFigure 4**. Number of months between the pre-pandemic CBCL assessment and start of the pandemic
**eFigure 5**. Number of months between the start of the pandemic and the mid-pandemic CBCL assessment
**eFigure 6**. Number of months between the pre-pandemic and mid-pandemic CBCL assessments
**eTable 1**. Description of ECHO cohorts included in the analytic sample
**eTable 2**. Frequency of youth categorized in the borderline or clinical range on CBCL broadband composites and DSM-5 subscales before and during the COVID-19 pandemic
**eTable 3**. Generalized linear mixed effects model estimating the impact of child ethnicity on change in child mental health (N=1229)
**eTable 4**. Generalized linear mixed effects model estimating the impact of child sex on the rate of change in child mental health (N=1229)
**eTable 5**. Generalized linear mixed-effects model estimating the impact of child age on change in child mental health (n=1229)
**eTable 6**. Model-based mean scores pre- and mid-pandemic and their difference (LS means), by child age
**eTable 7**. Model-based mean scores pre- and mid-pandemic and their difference (LS means), by poverty level
**eTable 8**. Model-based mean scores pre- and mid-pandemic and their difference (LS means), by child race
**eTable 9**. Model-based mean scores pre- and mid-pandemic and their difference (LS means), by CBCL threshold
**eTable 10**. Subgroup sample sizes for 3-way interaction models
**eTable 11**. Generalized linear mixed effects model estimating the 3-way interaction visit×age×CBCL threshold (N=1229)
**eTable 12**. Model-based mean scores pre- and mid-pandemic and their difference (LS means), by CBCL threshold*child age
**eTable 13**. Generalized linear mixed effects model estimating the 3-way interaction visit×sex×CBCL threshold (N=1229)
**eTable 14**. Model-based mean scores pre- and mid-pandemic and their difference (LS means), by CBCL threshold*child sex
**eTable 15**. Sensitivity analysis using continuous age variable in the generalized linear mixed-effects model estimating change in child mental health (n=1229)
**eTable 16**. Sensitivity analysis including time between pre- and mid-pandemic assessments variable in the generalized linear mixed-effects model estimating change in child mental health (n=1229)

data sharing statement **SUPPLEMENT 3**. Data Sharing Statement

collaborators **SUPPLEMENT 2**. Environmental influences on Child Health Outcomes Program Collaborators

## Figures and Tables

**Table 1.** Study Participant Characteristics vs Eligible Sample

Characteristic	Participants, No. (%)	
	Studysample (n = 1229)	Eligible sample (n = 5319)
Child age, mean (SD) [range], y		
Prepandemic	8.07 (1.83) [6–16]	9.34 (2.59) [5.67–17.99]^a^
During pandemic	10.68 (2.29) [7–17]	NA
Child sex	1229 (100.0)	5307 (99.8)
Male	604 (49.1)	2771 (52.1)
Female	625 (50.9)	2528 (47.5)
Ambiguous	0	8 (<1)
Missing	0	12 (<1)
Child race^b,c^	1210 (98.5)	4806 (90.4)
Black	388 (31.6)	1278 (24.0)
White	635 (51.7)	2476 (46.6)
Multiple races^d^	147 (12.0)	866 (16.3)
Other^e^	40 (3.3)	186 (3.5)
Missing	19 (1.5)	513 (9.6)
Child ethnicity^b^	1227 (99.8)	4916 (92.4)
Hispanic	118 (9.6)	660 (12.4)
Non-Hispanic	1109 (90.2)	4256 (80.0)
Missing	<5 (NA)	403 (7.6)
Caregiver’s highest level of education	1180 (96.0)	4143 (77.9)
Less than high school degree	20 (1.6)	182 (3.4)
High school degree or equivalent	89 (7.2)	586 (11.0)
Some college, associate degree, or trade school	248 (20.2)	889 (16.7)
Bachelor’s degree	333 (27.1)	1136(21.4)
Master’s degree and higher	490 (39.9)	1350 (25.4)
Missing	49 (4.0)	1176 (22.1)
Family income, percentage of Federal Poverty Level	1056 (85.9)	2485 (46.7)
≤130	197 (16.0)	518 (9.7)
>130–350	300 (24.4)	726 (13.6)
>350	559 (45.5)	1241 (23.3)
Missing	173 (14.1)	2834 (53.3)
Caregiver depression	1116 (91.0)	3136 (59.0)
T score		
Mean (SD) [range]	47.97 (8.17) [33.00–81.14]	48.47 (7.13) [33.00–76.04]
Median (IQR)	47.83 (38.28–54.1)	48.35 (43.13–53.75)
Missing	113 (9.2)	2183 (41.0)
Caregiver perceived stress	1215 (99.0)	3333 (63.0)
T score		
Mean (SD) [range]	48.59 (9.15) [22.37–82.18]	49.22 (8.81) [22.37–82.18]
Median (IQR)	48.25 (42.56–55.9)	49.10 (43.52–55.06)
Missing	14 (1.1)	1986 (37.3)

Abbreviation: NA, not applicable.

a Child age was based on age before and during pandemic Child Behavior Checklist assessment, and the eligible sample included children who did not have both time points, such that eligible sample age was calculated on the basis of the single assessment that occurred any time between January 1, 2015, and August 31, 2022.

b Child race and ethnicity were reported by the caregiver in accordance with National Institutes of Health (NIH) requirements related to reporting of individual-level participant data.

c For child race, caregivers were asked to select all that apply from the following categories, which reflect NIH reporting requirements: American Indian or Alaska Native, Asian Indian, Other Asian (which, if selected, expanded to include Chinese, Filipino, Japanese, Korean, Vietnamese, Other Asian, and do not know), Native Hawaiian or Other Pacific Islander, some other race, prefer not to answer, and do not know.

d Participants were categorized as multiracial if they selected more than 1 race for this question.

e Owing to small sample sizes and concerns about identifiability, children identified as American Indian or Alaska Native, Asian Indian, Other Asian, Native Hawaiian or Other Pacific Islander, or other race were combined into the “Other Race” category.

**Table 2.** Generalized Linear Mixed-Effects Model Estimating Change in Child Mental Health (N = 1229)

Parameter	β (95% CI)				
	Internalizing	Externalizing	Depression	Anxiety	ADHD
Intercept	5.15 (3.95 to 6.35)	6.00 (4.64 to 7.36)	1.40 (0.91 to 1.89)	2.13 (1.59 to 2.66)	3.45 (2.74 to 4.16)
Visit (during pandemic)	0.09 (−0.19 to 0.37)	−0.88 (−1.16 to −0.60)	0.22 (0.10 to 0.35)	−0.18 (−0.31 to −0.05)	−0.36 (−0.50 to −0.22)
Time between pandemic start and survey administration	0.02 (−0.02 to 0.06)	0.01 (−0.04 to 0.05)	0.01 (−0.01 to 0.03)	0.01 (−0.02 to 0.02)	0.01 (−0.01 to 0.04)
Family income, percentage of Federal Poverty Level					
≤130	0.84 (−0.16 to 1.83)	0.88 (−0.25 to 2.02)	0.36 (−0.02 to 0.75)	0.22 (−0.23 to 0.67)	−0.04 (−0.64 to 0.57)
130–350	0.58 (−0.12 to 1.28)	0.24 (−0.57 to 1.05)	0.23 (−0.05 to 0.50)	0.11 (−0.24 to 0.46)	0.07 (−0.35 to 0.48)
>350	0 [Reference]	0 [Reference]	0 [Reference]	0 [Reference]	0 [Reference]
Child age, y					
<12	−0.11 (−0.93 to 0.71)	0.38 (−0.55 to 1.30)	−0.16 (−0.47 to 0.15)	0.37 (−0.01 to 0.74)	0.21 (−0.27 to 0.69)
≥12	0 [Reference]	0 [Reference]	0 [Reference]	0 [Reference]	0 [Reference]
Child sex					
Female	0.24 (−0.28 to 0.75)	−1.26 (−1.82 to −0.68)	0.01 (−0.18 to 0.20)	0.06 (−0.18 to 0.29)	−1.02 (−1.31 to −0.72)
Male	0 [Reference]	0 [Reference]	0 [Reference]	0 [Reference]	0 [Reference]
Child race					
Black	−0.99 (−1.73 to −0.24)	−0.13 (−0.96 to 0.73)	−0.32 (−0.60 to −0.04)	−0.64 (−0.98 to −0.29)	−0.13 (−0.56 to 0.31)
Other race^a^	−0.27 (−1.02 to 0.47)	−0.50 (−1.34 to 0.33)	−0.14 (−0.41 to 0.14)	−0.20 (−0.55 to 0.14)	−0.44 (−0.87 to −0.01)
White	0 [Reference]	0 [Reference]	0 [Reference]	0 [Reference]	0 [Reference]
Child ethnicity					
Hispanic	−0.62 (−1.57 to 0.31)	−0.83 (−1.89 to 0.23)	−0.07 (−0.42 to 0.28)	−0.15 (−0.59 to 0.29)	−0.24 (−0.78 to 0.31)
Non-Hispanic	0 [Reference]	0 [Reference]	0 [Reference]	0 [Reference]	0 [Reference]
Caregiver educational attainment					
Less than high school	−0.79 (−2.99 to 1.42)	0.94 (−1.51 to 3.39)	−0.04 (−0.87 to 0.79)	−0.03 (−1.06 to 1.0)	1.42 (0.12 to 2.72)
High school degree or equivalent	−0.16 (−1.40 to 1.07)	1.70 (0.33 to 3.08)	0.08 (−0.39 to 0.55)	−0.09 (−0.65 to 0.48)	0.92 (0.18 to 1.65)
Some college, associate degree, or trade school	−0.29 (−1.14 to 0.56)	0.51 (−0.45 to 1.47)	−0.14 (−0.45 to 0.18)	−0.15 (−0.55 to 0.24)	0.80 (0.31 to 1.30)
Bachelor’s degree	−0.03 (−0.70 to 0.65)	0.51 (−0.24 to 1.26)	0.002 (−0.25 to 0.25)	−0.09 (−0.40 to 0.22)	0.67 (0.28 to 1.07)
Master’s, PhD, or professional degree	0 [Reference]	0 [Reference]	0 [Reference]	0 [Reference]	0 [Reference]
Caregiver depression	0.12 (0.08 to 0.16)	0.14 (0.10 to 0.18)	0.04 (0.02 to 0.05)	0.05 (0.03 to 0.07)	0.05 (0.03 to 0.07)
Caregiver perceived stress	0.09 (0.06 to 0.13)	0.09 (0.05 to 0.13)	0.03 (0.02 to 0.04)	0.03 (0.02 to 0.05)	0.05 (0.03 to 0.07)

Abbreviation: ADHD, attention-deficit/hyperactivity disorder.

a Owing to small sample sizes and concerns about identifiability, children identified as American Indian or Alaska Native, Asian Indian, Other Asian, Native Hawaiian or Other Pacific Islander, or other race were combined into the “Other Race” category.

**Table 3.** Generalized Linear Mixed-Effects Model Estimating the Impact of Poverty Level on Change in Child Mental Health (N = 1229)^a^

Model parameter	3 (95% CI)				
	Internalizing	Externalizing	Depression	Anxiety	ADHD
Intercept	5.03 (3.83 to 6.24)	5.83 (4.47 to 7.20)	1.33 (0.84 to 1.83)	2.09 (1.55 to 2.63)	3.26 (2.55 to 3.98)
Family income, percentage of Federal Poverty Level					
130 (n = 197)	1.24 (0.17 to 2.30)	1.34 (0.16 to 2.52)	0.59 (0.17 to 1.01)	0.29 (−0.19 to 0.78)	0.35 (−0.28 to 0.98)
>130–350 (n = 300)	0.71 (−0.07 to 1.49)	0.51 (−0.37 to 1.38)	0.29 (−0.03 to 0.61)	0.17 (−0.22 to 0.56)	0.42 (−0.03 to 0.87)
>350 (n = 559)	0 [Reference]	0 [Reference]	0 [Reference]	0 [Reference]	0 [Reference]
Visit	0.33 (−0.07 to 0.72)	−0.55 (−0.94 to −0.16)	0.35 (0.17 to 0.53)	−0.12 (−0.30 to 0.06)	−0.01 (−0.21 to 0.19)
Visit and poverty level					
130 (n = 197)	−0.80 (−1.56 to −0.03)	−0.91 (−1.67 to −0.15)	−0.45 (−0.79 to −0.10)	−0.14 (−0.49 to 0.21)	−0.76 (−1.14 to −0.37)
>130–350 (n = 300)	−0.26 (−0.94 to 0.42)	−0.52 (−1.20 to 0.16)	−0.13 (−0.44 to 0.19)	−0.11 (−0.43 to 0.20)	−0.68 (−1.04 to −0.33)
>350 (n = 559)	0 [Reference]	0 [Reference]	0 [Reference]	0 [Reference]	0 [Reference]

Abbreviation: ADHD, attention-deficit/hyperactivity disorder.

a Model was adjusted for time between pandemic start and survey administration, child age, child sex, child race, child ethnicity, caregiver educational attainment, and caregiver depression.

**Table 4.** Generalized Linear Mixed-Effects Model Estimating the Impact of Child Race on Change in Child Mental Health (N = 1229)^a^

Parameter	3 (95% CI)				
	Internalizing	Externalizing	Depression	Anxiety	ADHD
Intercept	4.99 (3.78 to 6.19)	5.88 (4.51 to 7.24)	1.31 (0.81 to 1.80)	2.07 (1.52 to 2.61)	3.34 (2.62 to 4.06)
Child race					
Black (n = 388)	−0.55 (−1.36 to 0.26)	0.11 (−0.79 to 1.01)	−0.08 (−0.39 to 0.23)	−0.53 (−0.90 to −0.16)	0.14 (−0.33 to 0.60)
Other race (n = 187)^b^	−0.15 (−1.0 to 0.70)	−0.18 (−1.11 to 0.75)	−0.10 (−0.44 to 0.23)	−0.07 (−0.46 to 0.33)	−0.35 (−0.83 to 0.13)
White (n = 635)	0 [Reference]	0 [Reference]	0 [Reference]	0 [Reference]	0 [Reference]
Visit	0.41 (0.03 to 0.80)	−0.64 (−1.02 to −0.26)	0.39 (0.22 to 0.57)	−0.07 (−0.25 to 0.11)	−0.16 (−0.36 to 0.04)
Visit and child race					
Visit and Black	−0.87 (−1.50 to −0.25)	−0.45 (−1.07 to 0.18)	−0.48 (−0.76 to −0.20)	−0.21 (−0.50 to 0.08)	−0.52 (−0.83 to −0.20)
Visit and other race	−0.25 (−1.06 to 0.56)	−0.64 (−1.45 to 0.16)	−0.07 (−0.43 to 0.29)	−0.27 (−0.64 to 0.11)	−0.18 (−0.59 to 0.24)
Visit and White	0 [Reference]	0 [Reference]	0 [Reference]	0 [Reference]	0 [Reference]

Abbreviation: ADHD, attention-deficit/hyperactivity disorder.

a Model was adjusted for time between pandemic start and survey administration, child age, child sex, child ethnicity, poverty level, caregiver educational attainment, and caregiver depression.

b Owing to small sample sizes and concerns about identifiability, children identified as American Indian or Alaska Native, Asian Indian, Other Asian, Native Hawaiian or Other Pacific Islander, or other race were combined into the “Other Race” category.

**Table 5.** Generalized Linear Mixed-Effects Model Estimating the Impact of Prepandemic CBCL Threshold Status on Change in Child Mental Health (N = 1229)^a^

Parameter	3 (95% CI)				
	Internalizing	Externalizing	Depression	Anxiety	ADHD
Intercept	4.19 (3.14 to 5.23)	4.80 (3.76 to 5.84)	1.11 (0.68 to 1.53)	1.73 (1.24 to 2.21)	2.96 (2.40 to 3.53)
CBCL threshold					
Normal	0 [Reference]	0 [Reference]	0 [Reference]	0 [Reference]	0 [Reference]
Borderline	5.79 (4.76 to 6.83)	7.32 (6.24 to 8.40)	1.66 (1.24 to 2.09)	2.35 (1.85 to 2.84)	3.72 (3.13 to 4.32)
Clinical	10.78 (9.84 to 11.72)	13.81 (12.83 to 14.79)	3.59 (3.21 to 3.98)	4.43 (3.98 to 4.89)	5.67 (5.12 to 6.21)
Visit	0.40 (0.10 to 0.70)	−0.29 (−0.57 to −0.01)	0.30 (0.17 to 0.44)	−0.06 (−0.20 to 0.08)	−0.16 (−0.31 to −0.01)
Visit and CBCL threshold					
Normal	0 [Reference]	0 [Reference]	0 [Reference]	0 [Reference]	0 [Reference]
Borderline	−0.98 (−2.09 to 0.13)	−2.85 (−3.92 to −1.78)	−0.21 (−0.72 to 0.29)	−0.43 (−0.95 to 0.09)	−1.28 (−1.85 to −0.72)
Clinical	−2.87 (−3.88 to −1.87)	−4.88 (−5.84 to −3.92)	−0.81 (−1.26 to −0.35)	−1.16 (−1.63 to −0.70)	−1.40 (−1.91 to −0.89)

Abbreviations: ADHD, attention-deficit/hyperactivity disorder; CBCL, Child Behavior Checklist.

a Model was adjusted for time between pandemic start and survey administration, child age, child sex, child race, child ethnicity, poverty level, caregiver educational attainment, and caregiver depression. See eTable 2 in Supplement 1 for borderline, clinical, and normal subgroup sample sizes by outcome.

## Key Points

### Question

How did the COVID-19 pandemic impact youth mental health?

### Findings

In this cohort study of 1229 US youths, the pandemic was associated with minor changes in youth mental health overall. However, youth entering the pandemic with prepandemic mental health problems experienced notable improvements across all outcomes, and lower-income and Black youth had small improvements in attention-deficit/hyperactivity disorder symptoms.

### Meaning

These findings suggest that not all youth responded to the COVID-19 pandemic in the same way; these differences are critical to understand for recovery and may yield novel insights into causes of youth mental health problems.
