# Supplementary material for: Longitudinal Changes in Youth Mental Health From Before to During the COVID-19 Pandemic
Source: JAMA Netw Open. Author manuscript; Available in PMC 2025 Feb 25. (PMC11856357; doi:10.1001/jamanetworkopen.2024.30198)
Supplement: supplement — eFigure 1. Participant flow diagram eFigure 2. Distribution of child ages at the pre-pandemic CBCL assessment eFigure 3. Distribution of child ages at the mid-pandemic CBCL assessment eFigure 4. Number of months between the pre-pandemic CBCL assessment and start of the pandemic eFigure 5. Number of months between the start of the pandemic and the mid-pandemic CBCL assessment eFigure 6. Number of months between the pre-pandemic and mid-pandemic CBCL assessments eTable 1. Description of ECHO cohorts included in the analytic sample eTable 2. Frequency of youth categorized in the borderline or clinical range on CBCL broadband composites and DSM-5 subscales before and during the COVID-19 pandemic eTable 3. Generalized linear mixed effects model estimating the impact of child ethnicity on change in child mental health (N=1229) eTable 4. Generalized linear mixed effects model estimating the impact of child sex on the rate of change in child mental health (N=1229) eTable 5. Generalized linear mixed-effects model estimating the impact of child age on change in child mental health (n=1229) eTable 6. Model-based mean scores pre- and mid-pandemic and their difference (LS means), by child age eTable 7. Model-based mean scores pre- and mid-pandemic and their difference (LS means), by poverty level eTable 8. Model-based mean scores pre- and mid-pandemic and their difference (LS means), by child race eTable 9. Model-based mean scores pre- and mid-pandemic and their difference (LS means), by CBCL threshold eTable 10. Subgroup sample sizes for 3-way interaction models eTable 11. Generalized linear mixed effects model estimating the 3-way interaction visit×age×CBCL threshold (N=1229) eTable 12. Model-based mean scores pre- and mid-pandemic and their difference (LS means), by CBCL threshold*child age eTable 13. Generalized linear mixed effects model estimating the 3-way interaction visit×sex×CBCL threshold (N=1229) eTable 14. Model-based mean scores pre- and mid-pandemic and their diff [file NIHMS2055771-supplement-supplement.pdf]

Supplemental Online Content

Blackwell CK, Wu G, Chandran A, et al; the Environmental influences on Child Health Outcomes Program Collaborators. Longitudinal changes in youth mental health from before to during the COVID-19 pandemic. *JAMA Netw Open*. 2024;7(8):e2430198. doi:10.1001/jamanetworkopen.2024.30198

eFigure 1. Participant flow diagram
eFigure 2. Distribution of child ages at the pre-pandemic CBCL assessment
eFigure 3. Distribution of child ages at the mid-pandemic CBCL assessment
eFigure 4. Number of months between the pre-pandemic CBCL assessment and start of the pandemic
eFigure 5. Number of months between the start of the pandemic and the mid-pandemic CBCL assessment
eFigure 6. Number of months between the pre-pandemic and mid-pandemic CBCL assessments
eTable 1. Description of ECHO cohorts included in the analytic sample
eTable 2. Frequency of youth categorized in the borderline or clinical range on CBCL broadband composites and DSM-5 subscales before and during the COVID-19 pandemic
eTable 3. Generalized linear mixed effects model estimating the impact of child ethnicity on change in child mental health (N=1229)
eTable 4. Generalized linear mixed effects model estimating the impact of child sex on the rate of change in child mental health (N=1229)
eTable 5. Generalized linear mixed-effects model estimating the impact of child age on change in child mental health (n=1229)
eTable 6. Model-based mean scores pre- and mid-pandemic and their difference (LS means), by child age
eTable 7. Model-based mean scores pre- and mid-pandemic and their difference (LS means), by poverty level
eTable 8. Model-based mean scores pre- and mid-pandemic and their difference (LS means), by child race
eTable 9. Model-based mean scores pre- and mid-pandemic and their difference (LS means), by CBCL threshold
eTable 10. Subgroup sample sizes for 3-way interaction models
eTable 11. Generalized linear mixed effects model estimating the 3-way interaction visit×age×CBCL threshold (N=1229)
eTable 12. Model-based mean scores pre- and mid-pandemic and their difference (LS means), by CBCL threshold*child age
eTable 13. Generalized linear mixed effects model estimating the 3-way interaction visit×sex×CBCL threshold (N=1229)

© 2024 Blackwell CK et al. *JAMA Network Open.*

eTable 14. Model-based mean scores pre- and mid-pandemic and their difference (LS means), by CBCL threshold*child sex
eTable 15. Sensitivity analysis using continuous age variable in the generalized linear mixed-effects model estimating change in child mental health (n=1229)
eTable 16. Sensitivity analysis including time between pre- and mid-pandemic assessments variable in the generalized linear mixed-effects model estimating change in child mental health (n=1229)
eReferences

This supplemental material has been provided by the authors to give readers additional information about their work.

eFigure 1. Participant flow diagram

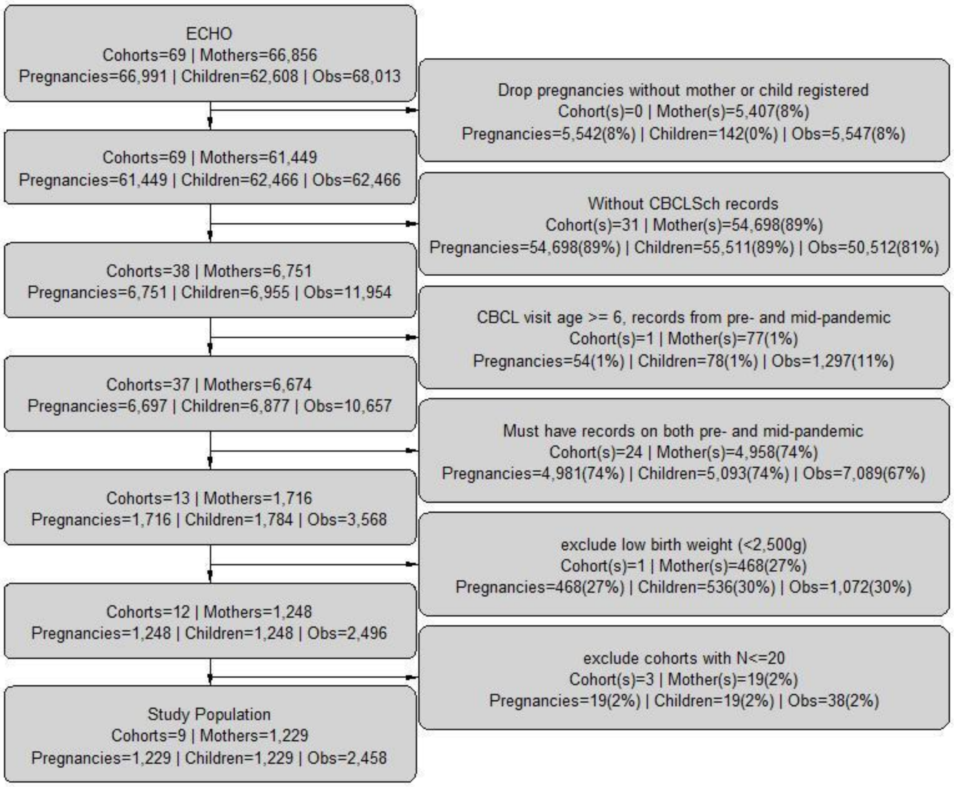

© 2024 Blackwell CK et al. *JAMA Network Open.*

eFigure 2. Distribution of child ages at the pre-pandemic CBCL assessment

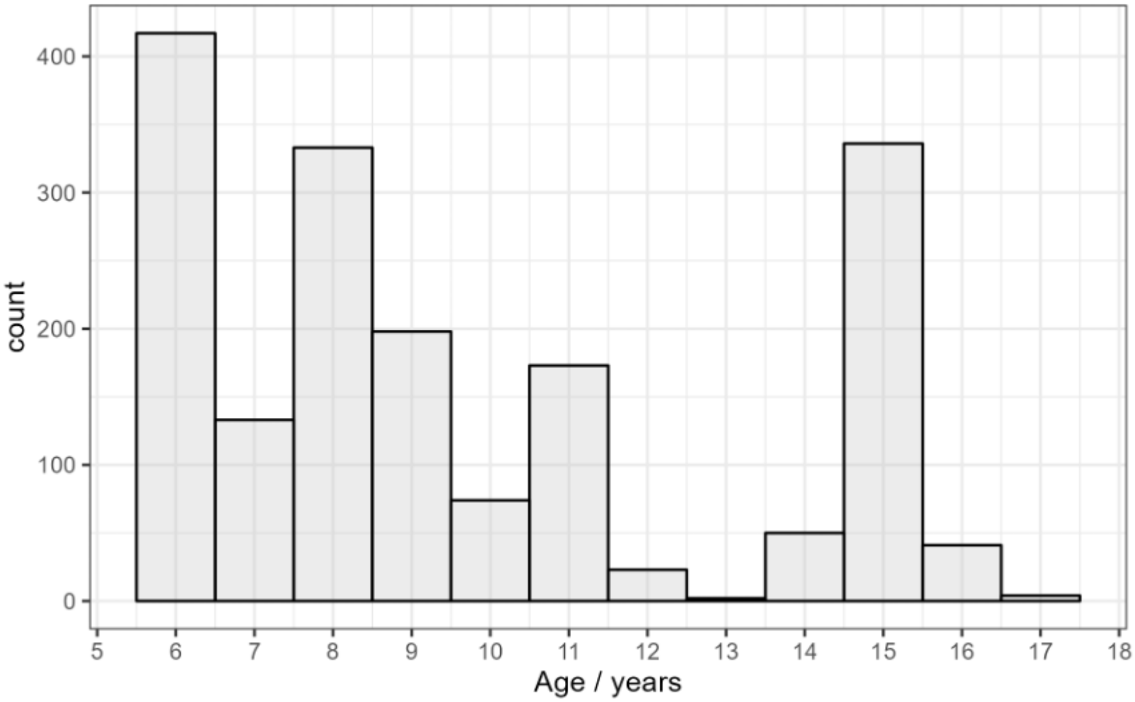

© 2024 Blackwell CK et al. *JAMA Network Open.*

eFigure 3. Distribution of child ages at the mid-pandemic CBCL assessment

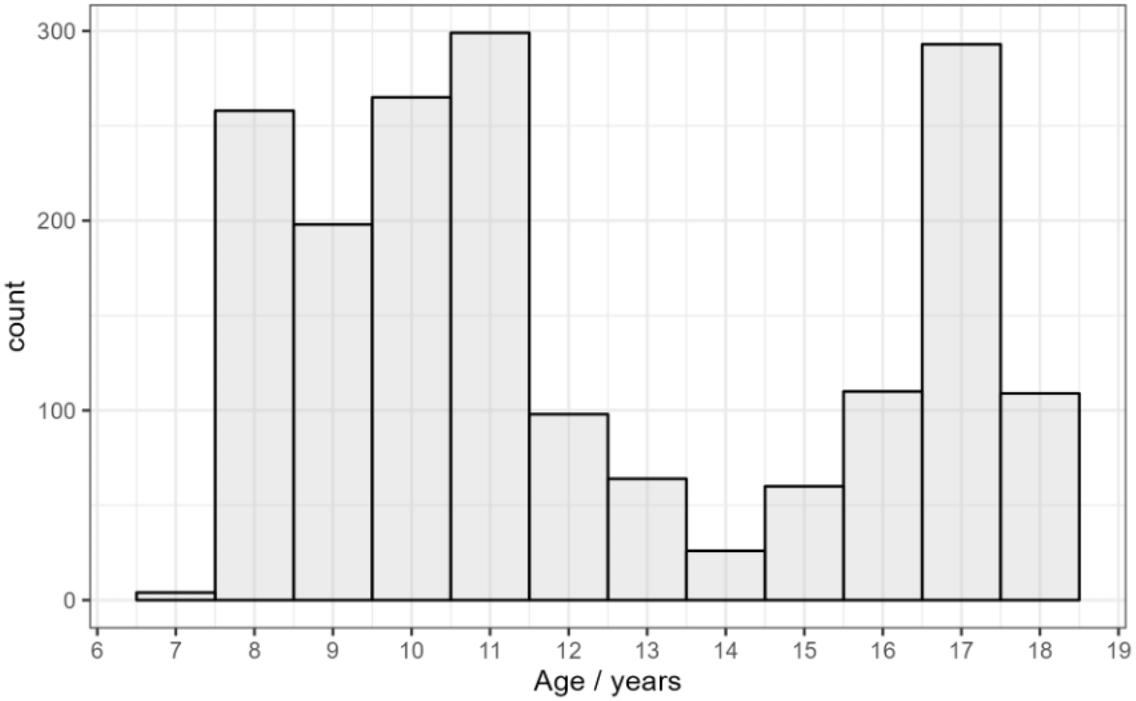

© 2024 Blackwell CK et al. *JAMA Network Open.*

eFigure 4. Number of months between the pre-pandemic CBCL assessment and start of the pandemic

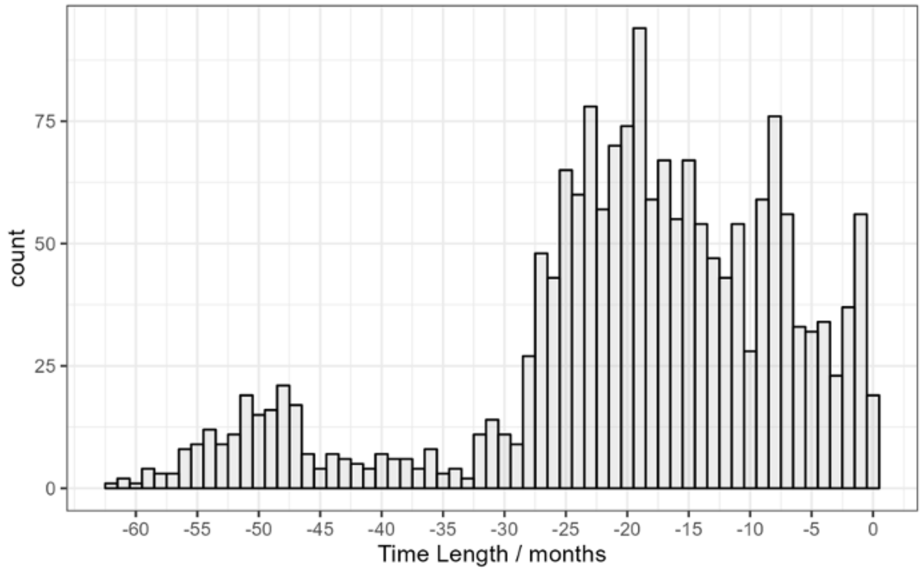

© 2024 Blackwell CK et al. *JAMA Network Open.*

eFigure 5. Number of months between the start of the pandemic and the mid-pandemic CBCL assessment

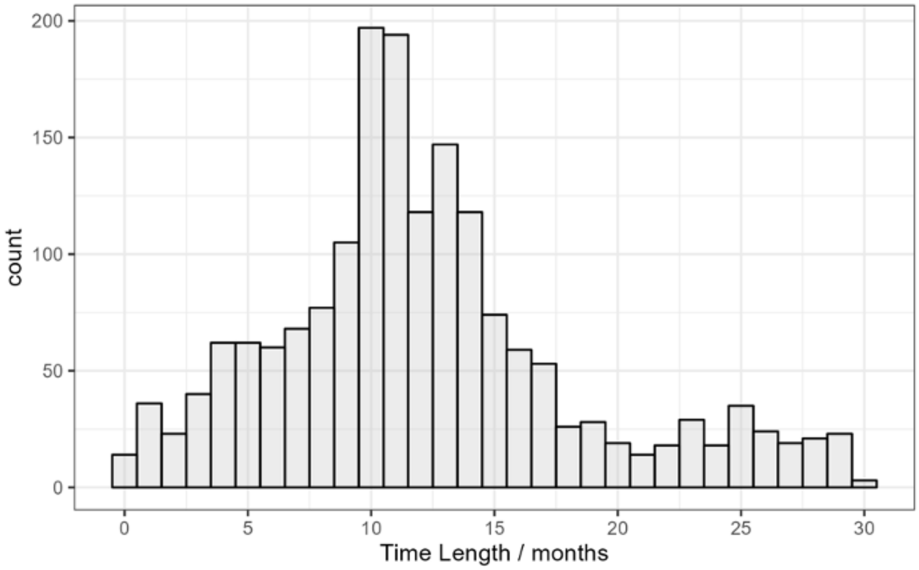

© 2024 Blackwell CK et al. *JAMA Network Open.*

eFigure 6. Number of months between the pre-pandemic and mid-pandemic CBCL assessments

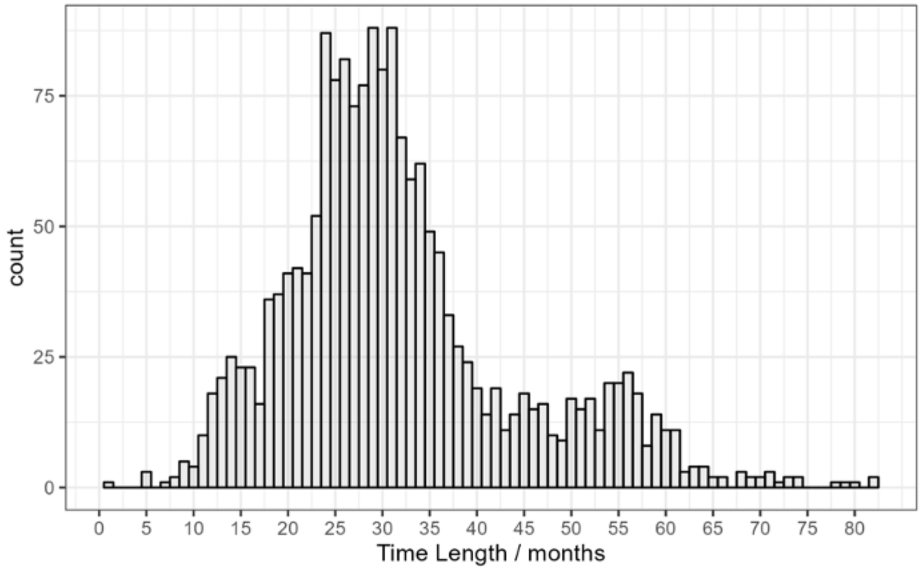

© 2024 Blackwell CK et al. *JAMA Network Open.*

eTable 1. Description of ECHO cohorts included in the analytic sample

Cohort	Study Overview	Recruitment Strategy	Inclusion/Exclusion Criteria
CANDLE[1-3] (n = 493)[a]	CANDLE enrolled 1,503 pregnant women who delivered between 2006-2011 at four hospitals in Memphis, TN, and 1,385 mother-child dyads are still under active follow-up. The original study goal was to understand early-life predictors of child socioemotional and neurocognitive development through age 3, with additional funding in 2011 allowing for follow-up of CANDLE children between ages 4-6, for atopy and asthma assessment and collection of environmental, cognitive, and socioemotional data; 1,157 mother-child dyads were assessed at this time point. The demographics of eligible, active CANDLE mothers reflect the demographics of Shelby County, TN. The cohort is primarily African American (63%) with the majority making less than $50,000 per year (64%). The original study included up to 15 clinical, home, and phone visits (pregnancy-age 3 years), resulting in rich characterization of maternal exposures during pregnancy and maternal and child experiences. Retention and follow-up of active CANDLE participants (N=1,385) has been strong, with follow-up rates of 80% at ages 1 and 2, 74% at age 3, and 84% at age 4. The cohort is actively being retained, and consents include permission to contact families through child age 18.	Participants were recruited from 2 sources in Memphis, TN from 2006-2011. The first source was from the Shelby County safety net obstetrical clinics at the Regional Medical Center in Memphis. This effort recruited 344 pregnant women from the most disadvantaged segment of Shelby County. The second recruitment wave focused on women residing in Shelby County. Most these participants were recruited through flyers provided by local OB Partner medical practices, television and radio advertisements, and directed mailings to targeted zip codes.	**Inclusion:** Shelby County TN resident; Pregnancy 16-28 weeks gestation (at enrollment); Between ages of 16 and 40; Speak and understand English; Singleton pregnancy; Low-risk pregnancy; Plans to deliver at one of five participating health care settings in Shelby Co, TN.<br><br>**Exclusion:** Less than 16 years of age or greater than 40 years of age; Multiple gestation in the current pregnancy; Chronic hypertension or vascular disease requiring therapy; Maternal red cell allommunization, except Rh factor; Hemoglobinopathy including sickle cell trait and severe iron deficiency anemia (hemoglobin < 9); Insulin dependent diabetes; Appreciable renal or cardiopulmonary disease; Prolapsed or ruptured membranes; Oligohydramnios; Complete placenta previa; refused consent, endocrine disease, collagen disease, active or chronic hepatitis, renal disease, pulmonary or heart disease requiring therapeutic medication or limitation of physical activity (except for mitral valve prolapse or asthma requiring only occasional medication), major fetal anomaly, infection with HIV, delivery or prenatal care outside clinical center

© 2024 Blackwell CK et al. *JAMA Network Open.*

Cohort	Study Overview	Recruitment Strategy	Inclusion/Exclusion Criteria
TIDES[1, 4]<br>(n = 381)[a]	TIDES is a prenatal cohort study originally focused on examining in utero phthalate exposure in relation to sex specific genital outcomes in infants. It has now expanded to examine sex specific neurobehavior in children.	Recruited from prenatal clinics from 2010-2012 and 2016+ at University of Minnesota, University of Washington, University of California - San Francisco, and University of Rochester.	**Inclusion:** <13 weeks pregnant; >18 years of age; Able to read/write in English; Planned to deliver at a study hospital; Pregnancy was not medically threatened.<br><br>**Exclusion:** Any mother who was taking hormone (estrogen/testosterone) supplements
Early Growth and Development Study[5]<br>(n = 118)[a]	The Early Growth and Development Study (EGDS) is a longitudinal prospective adoption study of biological parents, adoptive parents, and adopted children. The sample includes a set of 361 domestically adopted children who were assessed beginning at age 9 months. In addition, the biological parents were assessed at five different time points following the placement. The study design allows for exploration of the relationship between family environment and heredity in child development. The primary study aims are to examine how family, peer and contextual processes affect children's adjustment, and to examine their interplay (mediation, moderation) with genetic influences.	Participants were recruited from 2003-2006 through domestic adoption agencies located throughout the United States following the birth of the child. The study was conducted through a collaboration of various institutions since inception, including The Pennsylvania State University, the University of California at Davis, Yale University, the Oregon Social Learning Center, George Washington University, and the University of Oregon. Adoption agencies were located in Oregon, Washington, Texas, Maryland, Pennsylvania, Virginia, New Jersey, California, Utah, and Texas.	**Inclusion:** Domestic adoption placement; placement occurred within 3 months postpartum; the infant was placed with an adoptive family that was not biologically related to the child; birth and adoptive parents were able to understand English at the 8th-grade level; birth and adoptive family both separately agreed to participate.<br><br>**Exclusion:** Any known major medical conditions, such as extreme prematurity or extensive medical surgeries.

© 2024 Blackwell CK et al. *JAMA Network Open.*

Cohort	Study Overview	Recruitment Strategy	Inclusion/Exclusion Criteria
Early Growth and Development Study – Cohort II[5] (n = 64)[a]	The Early Growth and Development Study (EGDS) – Cohort II is a longitudinal prospective adoption study of biological parents, adoptive parents, and adopted children. The sample includes a set of 200 domestically adopted children who were assessed beginning at age 9 months. In addition, the biological parents were assessed at three different time points following the placement. The study design allows for exploration of the relationship between family environment and heredity in child development. The primary study aims are to examine how family, peer and contextual processes affect children's adjustment, and to examine their interplay (mediation, moderation) with genetic influences.	Participants were recruited from 2007-2010 through domestic adoption agencies located throughout the United States following the birth of the child. The study was conducted through a collaboration of various institutions since inception including The Pennsylvania State University, the University of California, Yale University, the Oregon Social Learning Center, George Washington University, and the University of Oregon. Agencies were located in Maryland, Pennsylvania, Minnesota, California, Illinois, Utah, Oregon, Washington, and Florida.	**Inclusion:** Domestic adoption placement; placement occurred within 3 months postpartum; the infant was placed with an adoptive family that was not biologically related to the child; birth and adoptive parents were able to understand English at the 8th-grade level; birth and adoptive family both separately agreed to participate.<br><br>**Exclusion:** Any known major medical conditions, such as extreme prematurity or extensive medical surgeries.

© 2024 Blackwell CK et al. *JAMA Network Open.*

Cohort	Study Overview	Recruitment Strategy	Inclusion/Exclusion Criteria
Early Parenting of Children Study[5] (n = 48)[a]	The Early Parenting of Children Study (EPoCh) is an extension study of the Early Growth and Development Study (EGDS) which utilizes a naturalistic human cross-fostering design to advance the understanding of childhood pathways to risk for drug use by identifying nuances in the rearing environment that are associated with child risk behaviors and competencies. In this quasi-experimental study, the sample consists of 200 sibling pairs in which one sibling was reared from birth with an adoptive family, and the other sibling was reared from birth by the biological mother of the sibling pair. Similar to non-human cross-fostering studies, this design allows for the differentiation of the effects of the rearing environment from effect of shared genes between parent and child.	Recruitment of 200 7-year-old siblings who were reared by the biological families of children from the original EGDS and EGDS-II cohorts occurred from 2014-2017.	**Inclusion:** Birth parent participated in Early Growth and Development Study (EGDS); Rearing a child who is age 7.<br><br>**Exclusion:** Birth parent did not participant in EGDS; no siblings
BAMBAM[6] (n = 48)[a]	The Brown university Assessment of Myelination and Behavioral development Across Maturation (BAMBAM) Study is an accelerated longitudinal study of a large community cohort of healthy children in and around the Providence, RI area. Half of the cohort was enrolled between ages 2-8 months, and half was enrolled between the ages of 2-4 years. Major project aims are investigating typical brain and neuropsychological development.	Recruitment occurred between 2010-2017 in Providence, RI using pamphlets in pediatrician offices, online webpages, radio ads, and advertisement in the general population.	**Inclusion:** Healthy children; No metal in body (MR safe for MRI component of study); No diagnosis of ADHD; No older than 12 years<br><br>**Exclusion:** Children with major risk factors for brain abnormalities; In utero alcohol, cigarette or illicit substance exposure; Preterm (<37wks gestation) birth; SGA or less than 1500g; Fetal ultrasound abnormalities; Complicated pregnancy including preeclampsia, high blood pressure, and GDM; APGAR scores <8; NICU admission; Neurological disorder (e.g., head injury, epilepsy); Psychiatric or learning

© 2024 Blackwell CK et al. *JAMA Network Open.*

Cohort	Study Overview	Recruitment Strategy	Inclusion/Exclusion Criteria
			disorder in the infant, parents or siblings (such as medicated depression)
Columbia Center for Children's Health (CCCEH) Sibling Study[7] (n = 29)[a]	Beginning in March 2008, pregnant women enrolled in the CCCEH Mothers and Newborns Study[8] were invited to participate in CCCEH's Sibling Study. Similar to the parent study, women were enrolled if they had a prenatal visit by the 20th week of pregnancy. The age range of the women at the start of this study was 27-44 years.	From 2008-2017, mothers enrolled in the Mothers and Newborns cohort were asked if they were pregnant, and if so, if they were willing to participate in the Sibling Study.	**Inclusion:** Enrollment in the CCCEH Mothers and Newborns Cohort; Enrollment in a prenatal clinic by the 20th week of pregnancy<br><br>**Exclusion:** Active smoking; Illicit drug use
GAPPS[1, 9] (n = 27)[a]	In 2007, the Seattle Children's Hospital launched the Global Alliance to Prevent Prematurity and Stillbirth (GAPPS). The purpose of the GAPPS Repository is to establish a data and tissue biobank to be used to research normal and abnormal pregnancies, including how pregnancy affects maternal and child health after delivery. Data and specimens come to the Repository through a network of collection sites that have each received approval for this project by their local IRBs and Ethics Committees and training in standardized collection procedures. Hospitals participating in the network as collection sites have racial/ethnic and socioeconomically diverse populations, high preterm birth rates and geographic and environmental differences.	Initial contact for study recruitment occurred between 2009-2017 as part of scheduling a prenatal visit, through introductory mailings, in the clinical setting, antepartum floors, at the time of delivery, or up to day seven postpartum in Seattle, WA and Yakima, WA.	**Inclusion:** Pregnant women 14 years of age or older; Women 18 years of age or older who are no longer pregnant, but have delivered less than or equal to 7 days of enrollment.<br><br>**Exclusion:** <18 who is not medically or legally emancipated, without parental consent; Anyone who is unable to provide informed consent.; Received narcotics in the 12 hours prior to being asked to provide consent.; In active labor by their physician's standards; In preterm labor ≤21 0/7 weeks gestation; Multiple gestations pregnancy

© 2024 Blackwell CK et al. *JAMA Network Open.*

Cohort	Study Overview	Recruitment Strategy	Inclusion/Exclusion Criteria
Programming of Intergenerational Stress of Mechanisms[10] (n = 21)[a]	The PRISM cohort is a longitudinal pregnancy cohort (N=350) to test the impact of maternal and child pre- and postnatal stress exposures on maternal prenatal and child stress regulation and on child health outcomes in infancy and early childhood, with a focus on respiratory and neurodevelopmental health. Additional funding allowed for expansion of the cohort to >2,500, biobanking of placenta/cord blood and umbilical vessels for epigenomics, and satellite-based modeling air assessments.	Between March 2011 to August 2013, women were recruited from prenatal clinics at the Brigham and Women's Hospital, Beth Israel Deaconess Medical Center, and the East Boston Neighborhood Health Center in Boston, MA. In August 2012, recruitment expanded to the obstetrics clinic at the Mount Sinai Hospital in New York, NY. Women were recruited in mid-pregnancy (23.1+6.1 weeks gestation) through 2017.	**Inclusion:** Mother 18 years or older at recruitment in pregnancy; Single gestation pregnancy; Mother English or Spanish speaking.<br><br>**Exclusion:** At enrollment, endorsement of drinking >= 7 alcoholic drinks/week prior to pregnancy recognition; At enrollment, endorsement of any drinking after pregnancy recognition; Maternal or child chronic health conditions that would impede study participation.

[a]Cohort sample sizes reflect the number of participants included in the analytic sample.

© 2024 Blackwell CK et al. *JAMA Network Open.*

eTable 2. Frequency of youth categorized in the borderline or clinical range on CBCL broadband composites and DSM-5 subscales before and during the COVID-19 pandemic

	Pre-pandemic	During Pandemic
Internalizing Problems		
Borderline N(%)	86 (7%)	60 (5%)
Clinical N(%)	101 (8%)	119 (10%)
Externalizing Problems		
Borderline N(%)	73 (6%)	66 (5%)
Clinical N(%)	101 (8%)	73 (6%)
DSM-5 Depressive Problems*		
Borderline N(%)	42 (4%)	64 (5%)
Clinical N(%)	26 (2%)	45 (4%)
DSM-5 Anxiety Problems		
Borderline N(%)	61 (5%)	63 (5%)
Clinical N(%)	39 (3%)	52 (4%)
DSM-5 ADHD Problems		
Borderline N(%)	64 (5%)	46 (4%)
Clinical N(%)	45 (4%)	57 (5%)

Abbreviations: ADHD=attention deficit/hyperactivity disorder; CBCL=Child Behavior Checklist; DSM-5=Diagnostic and Statistical Manual – 5th edition.

© 2024 Blackwell CK et al. *JAMA Network Open.*

eTable 3. Generalized linear mixed effects model estimating the impact of child ethnicity on change in child mental health (N=1229)

	Internalizing	Externalizing	Depression	Anxiety	ADHD
**Model parameter**	**β (95% CI)**	**β (95% CI)**	**β (95% CI)**	**β (95% CI)**	**β (95% CI)**
Intercept	5.13 (3.93, 6.33)	6.01 (4.65, 7.37)	1.40 (0.91, 1.89)	2.12 (1.58, 2.65)	3.44 (2.73, 4.16)
Child ethnicity					
Hispanic	-0.40 (-1.46, 0.66)	-0.97 (-2.13, 0.19)	-0.05 (-0.47, 0.37)	-0.03 (-0.53, 0.48)	-0.18 (-0.78, 0.43)
Non-Hispanic (ref)					
Visit	0.14 (-0.16, 0.43)	-0.91 (-1.20, -0.62)	0.23 (0.10, 0.36)	-0.16 (-0.29, -0.02)	-0.35 (-0.50, -0.20)
Visit×child ethnicity					
Visit×Hispanic	-0.45 (-1.41, 0.51)	0.28 (-0.68, 1.23)	-0.05 (-0.48, 0.39)	-0.24 (-0.69, 0.21)	-0.11 (-0.61, 0.38)
Visit×Non-Hispanic (ref)					

Abbreviations: ADHD=attention deficit/hyperactivity disorder; ref=reference category.
Model was adjusted for time between pandemic start and survey administration, child age, child sex, child race, poverty level, caregiver educational attainment, and caregiver depression. No least squares means were estimated as no significant differences were observed by child ethnicity. $N_{Hispanic}$ = 118, $N_{Non\text{-}Hispanic}$ = 1109.

© 2024 Blackwell CK et al. *JAMA Network Open.*

eTable 4. Generalized linear mixed effects model estimating the impact of child sex on change in child mental health (N=1229)

	Internalizing	Externalizing	Depression	Anxiety	ADHD
**Model parameter**	**β (95% CI)**	**β (95% CI)**	**β (95% CI)**	**β (95% CI)**	**β (95% CI)**
Intercept	5.24 (4.03, 6.44)	6.14 (4.78, 7.51)	1.46 (0.97, 1.96)	2.12 (1.58, 2.66)	3.49 (2.77, 4.21)
Child sex					
Female	0.07 (-0.52, 0.65)	-1.54 (-2.17, -0.90)	-0.11 (-0.34, 0.12)	0.08 (-0.20, 0.35)	-1.10 (-1.43, -0.77)
Male (ref)					
Visit	-0.08 (-0.48, 0.32)	-1.17 (-1.56, -0.77)	0.10 (-0.08, 0.28)	-0.16 (-0.34, 0.02)	-0.44 (-0.64, -0.24)
Visit×child sex					
Visit×female	0.34 (-0.21, 0.90)	0.56 (0.01, 1.11)	0.24 (-0.01, 0.4)	-0.04 (-0.30, 0.22)	0.16 (-0.13, 0.44)
Visit×male (ref)					

Abbreviations: ADHD=attention deficit/hyperactivity disorder; ref=reference category.
Model was adjusted for time between pandemic start and survey administration, child age, child race, child ethnicity, poverty level, caregiver educational attainment, and caregiver depression.
No least squares means were estimated as no significant differences were observed by child sex. N_{female} = 625, N_{male} = 604.

© 2024 Blackwell CK et al. *JAMA Network Open.*

eTable 5. Generalized linear mixed-effects model estimating the impact of child age on change in child mental health (n=1229)

	Internalizing	Externalizing	Depression	Anxiety	ADHD
**Model parameter**	**β (95% CI)**	**β (95% CI)**	**β (95% CI)**	**β (95% CI)**	**β (95% CI)**
Intercept	4.86 (3.63, 6.09)	5.85 (4.47, 7.24)	1.21 (0.70, 1.72)	2.18 (1.63, 2.73)	3.42 (2.70, 4.15)
Child age, y					
< 12	0.27 (-0.61, 1.16)	0.57 (-0.41, 1.55)	0.09 (-0.26, 0.44)	0.30 (-0.11, 0.71)	0.24 (-0.27, 0.75)
≥ 12 (ref)					
Visit	0.67 (0.09, 1.24)	-0.59 (-1.17, -0.02)	0.58 (0.32, 0.84)	-0.28 (-0.55, -0.01)	-0.31 (-0.61, -0.02)
Visit×child age, y					
< 12	-0.75 (-1.40, -0.09)	-0.38 (-1.03, 0.28)	-0.47 (-0.76, -0.17)	0.13 (-0.18, 0.43)	-0.06 (-0.40, 0.28)
≥ 12 (ref)					

Abbreviations: ADHD = attention deficit/hyperactivity disorder; ref=reference category.
Model was adjusted for time between pandemic start and survey administration, child sex, child race, child ethnicity, poverty level, caregiver educational attainment, and caregiver depression.
$N_{<12}$ = 941, $N_{>=12}$ = 248.

© 2024 Blackwell CK et al. *JAMA Network Open.*

eTable 6. Model-based mean scores pre- and mid-pandemic and their difference (LS means), by child age group

	Internalizing			Externalizing			Depression			Anxiety			ADHD		
**Child Age Group**	***Pre***	***Mid***	***Diff***	***Pre***	***Mid***	***Diff***	***Pre***	***Mid***	***Diff***	***Pre***	***Mid***	***Diff***	***Pre***	***Mid***	***Diff***
< 12	4.95	4.87	-0.08	6.36	5.39	-0.97	1.41	1.53	0.12	2.24	2.09	-0.15	3.77	3.40	-0.37
≥12 (ref)	4.67	5.34	0.67	5.79	5.20	-0.59	1.32	1.90	0.58	1.94	1.66	-0.28	3.53	3.22	-0.31

Abbreviations: ADHD=attention deficit/hyperactivity disorder; diff=difference; LS=least squares; mid=mid-pandemic; pre=pre-pandemic; ref=reference category.
Model was adjusted for time between pandemic start and survey administration, child sex, child race, child ethnicity, poverty level, caregiver educational attainment, and caregiver depression.
$N_{<12}$ = 941, $N_{>=12}$ = 248.

© 2024 Blackwell CK et al. *JAMA Network Open.*

eTable 7. Model-based mean scores pre- and mid-pandemic and their difference (LS means), by poverty level

	Internalizing			Externalizing			Depression			Anxiety			ADHD		
	Pre	***Mid***	***Diff***	***Pre***	***Mid***	***Diff***	***Pre***	***Mid***	***Diff***	***Pre***	***Mid***	***Diff***	***Pre***	***Mid***	***Diff***
≤130%	5.56	5.09	-0.47	6.92	5.46	-1.46	1.76	1.66	-0.09	2.22	1.96	-0.26	3.82	3.05	-0.76
130-350%	5.04	5.10	0.07	6.09	5.02	-1.07	1.46	1.69	0.22	2.10	1.86	-0.23	3.89	3.19	-0.69
>350%	4.33	4.66	0.33	5.59	5.03	-0.55	1.17	1.52	0.35	1.93	1.81	-0.12	3.47	3.46	-0.01

Abbreviations: ADHD=attention deficit/hyperactivity disorder; diff=difference; LS=least squares; mid=mid-pandemic; pre=pre-pandemic; ref=reference category.
Model was adjusted for time between pandemic start and survey administration, child age, child sex, child race, child ethnicity, caregiver educational attainment, and caregiver depression.
$N_{<=130\%}$ = 197, $N_{>130-350\%}$ = 300, $N_{>350\%}$ = 559.

© 2024 Blackwell CK et al. *JAMA Network Open.*

eTable 8. Model-based mean scores pre- and mid-pandemic and their difference (LS means), by child race

	Internalizing			Externalizing			Depression			Anxiety			ADHD		
	Pre	***Mid***	***Diff***	***Pre***	***Mid***	***Diff***	***Pre***	***Mid***	***Diff***	***Pre***	***Mid***	***Diff***	***Pre***	***Mid***	***Diff***
Black	4.63	4.17	-0.46	6.32	5.23	-1.08	1.42	1.33	-0.09	1.76	1.48	-0.28	3.88	3.20	-0.68
Other Race	5.03	5.19	0.16	6.03	4.75	-1.28	1.40	1.72	0.32	2.23	1.89	-0.33	3.40	3.06	-0.34
White	5.18	5.59	0.41	6.21	5.57	-0.64	1.50	1.89	0.39	2.29	2.22	-0.07	3.75	3.58	-0.16

Abbreviations: ADHD=attention deficit/hyperactivity disorder; diff=difference; LS=least squares; mid=mid-pandemic; pre=pre-pandemic; ref=reference category.
Model was adjusted for time between pandemic start and survey administration, child age, child sex, child ethnicity, poverty level, caregiver educational attainment, and caregiver depression.
N_{Black} = 388, $N_{Other\ Race}$ = 187, N_{White} = 635.

© 2024 Blackwell CK et al. *JAMA Network Open.*

eTable 9. Model-based mean scores pre- and mid-pandemic and their difference (LS means), by CBCL threshold

	Internalizing			Externalizing			Depression			Anxiety			ADHD		
**CBCL Threshold**	***Pre***	***Mid***	***Diff***	***Pre***	***Mid***	***Diff***	***Pre***	***Mid***	***Diff***	***Pre***	***Mid***	***Diff***	***Pre***	***Mid***	***Diff***
Borderline	9.15	8.57	-0.58	11.50	8.36	-3.14	2.60	2.69	0.09	3.79	3.30	-0.49	6.53	5.08	-1.45
Clinical	14.14	11.67	-2.47	17.99	12.83	-5.17	4.53	4.03	-0.51	5.88	4.65	-1.22	8.48	6.91	-1.56
Normal	3.36	3.76	0.40	4.18	3.90	-0.29	0.94	1.24	0.30	1.45	1.39	-0.06	2.81	2.65	-0.16

Abbreviations: ADHD=attention deficit/hyperactivity disorder; diff=difference; LS=least squares; mid=mid-pandemic; pre=pre-pandemic; ref=reference category.
See eTable 2 for borderline, clinical, and normal subgroup sample sizes by outcome.

© 2024 Blackwell CK et al. *JAMA Network Open.*

eTable 10. Subgroup sample sizes for 3-way interaction models

CBCL Threshold	Child Characteristic	Sample Size (n)
**Borderline**		
	Female	31
	Male	48
	Age<12	58
	Age>=12	21
**Clinical**		
	Female	41
	Male	59
	Age<12	77
	Age>=12	23
**Normal**		
	Female	553
	Male	497
	Age<12	806
	Age>=12	244

Abbreviations: CBCL=Child Behavior Checklist.

© 2024 Blackwell CK et al. *JAMA Network Open.*

eTable 11. Generalized linear mixed effects model estimating the 3-way interaction visit×age×CBCL threshold (N=1229)

	Internalizing	Externalizing
**Parameter**	**β (95% CI)**	**β (95% CI)**
Intercept	3.94 (2.84, 5.03)	4.62 (3.52, 5.72)
Child age, y		
< 12	0.003 (-0.79, 0.80)	0.29 (-0.54, 1.11)
≥ 12 (ref)		
CBCL threshold		
Borderline	6.76 (4.78, 8.75)	6.52 (4.44, 8.60)
Clinical	9.33 (7.43, 11.24)	14.74 (12.75, 16.74)
Normal (ref)		
Age×CBCL threshold		
Age×borderline	-1.30 (-3.61, 1.02)	1.12 (-1.30, 3.54)
Age×clinical	1.90 (-0.27, 4.06)	-1.19 (-3.46, 1.07)
Age×normal (ref)		
Visit	0.90 (0.29, 1.52)	0.15 (-0.44, 0.74)
Visit×child age, y		
Visit×age <12	-0.65 (-1.35, 0.05)	-0.57 (-1.25, 0.10)
Visit×age ≥12 (ref)		
Visit×CBCL threshold		
Visit×borderline	-2.56 (-4.72, -0.40)	-3.87 (-5.95, -1.79)
Visit×clinical	-0.4 (-2.47, 1.67)	-5.48 (-7.47, -3.48)
Visit×normal (ref)		
Visit×age×CBCL threshold		
Visit×age×borderline	2.10 (-0.41, 4.62)	1.35 (-1.07, 3.78)
Visit×age×clinical	-3.22 (-5.58, -0.86)	0.78 (-1.50, 3.05)
Visit×age×normal (ref)		

Abbreviations: ref=reference category.
Model was adjusted for time between pandemic start and survey administration, child sex, child race, child ethnicity, poverty level, caregiver educational attainment, and caregiver depression.
See eTable 10 for subgroup sample sizes.

© 2024 Blackwell CK et al. *JAMA Network Open.*

eTable 12. Model-based mean scores pre- and mid-pandemic and their difference (LS means), by CBCL threshold*child age

	**Internalizing**			**Externalizing**		
**CBCL Threshold, Age**	***Pre***	***Mid***	***Diff***	***Pre***	***Mid***	***Diff***
Borderline, < 12 y	8.74	8.54	-0.20	11.90	8.97	-2.94
Borderline, ≥ 12 y	10.03	8.38	-1.65	10.50	6.78	-3.72
Clinical, <12 y	14.51	11.14	-3.37	17.82	12.70	-5.12
Clinical, ≥ 12 y	12.60	13.11	0.50	18.72	13.39	-5.33
Normal, < 12 y	3.27	3.52	0.25	0.85	1.53	0.68
Normal, ≥ 12 y	3.27	4.17	0.90	0.88	1.07	0.19

Abbreviations: CBCL=Child Behavior Checklist; diff=difference; LS=least squares; mid=mid-pandemic; pre=pre-pandemic; ref=reference category.
Model was adjusted for time between pandemic start and survey administration, child sex, child race, child ethnicity, poverty level, caregiver educational attainment, and caregiver depression.
See eTable 10 for subgroup sample sizes.

© 2024 Blackwell CK et al. *JAMA Network Open.*

eTable 13. Generalized linear mixed effects model estimating the 3-way interaction visit×sex×CBCL threshold (N=1229)

	Internalizing	Externalizing
**Parameter**	**β (95% CI)**	**β (95% CI)**
Intercept	4.37 (3.31, 5.42)	4.81 (3.76, 5.86)
Child sex		
Female	0.342 (-0.20, 0.88)	-0.76 (-1.32, -0.19)
Male (ref)		
CBCL threshold		
Borderline	5.65 (4.33, 6.97)	7.00 (5.62, 8.38)
Clinical	9.52 (8.30, 10.75)	14.58 (13.30, 15.86)
Normal (ref)		
Child sex*CBCL threshold		
Female×borderline	0.28 (-1.80, 2.35)	0.84 (-1.33, 3.01)
Female×clinical	2.96 (1.10, 4.81)	-1.86 (-3.80, 0.08)
Male×normal (ref)		
Visit	-0.22 (-0.67, 0.23)	-0.94 (-1.38, -0.49)
Visit×child sex		
Visit×female	0.22 (-0.37, 0.81)	0.32 (-0.25, 0.88)
Visit×male (ref)		
Visit×CBCL threshold		
Visit×borderline	-2.27 (-3.71, -0.84)	-3.41 (-4.79, -2.03)
Visit×clinical	-1.70 (-3.01, -0.38)	-4.24 (-5.50, -2.97)
Visit×normal (ref)		
Visit×female×CBCL threshold		
Visit×female×borderline	3.35 (1.09, 5.61)	1.53 (-0.65, 3.70)
Visit×female×clinical	-2.78 (-4.80, -0.76)	-1.48 (-3.43, 0.46)
Visit×male×normal (ref)		

Abbreviations: ref=reference category.
Model was adjusted for time between pandemic start and survey administration, child age, child race, child ethnicity, poverty level, caregiver educational attainment, and caregiver depression.
See eTable 10 for subgroup sample sizes.

© 2024 Blackwell CK et al. *JAMA Network Open.*

eTable 14. Model-based mean scores pre- and mid-pandemic and their difference (LS means), by CBCL threshold*child sex

	Internalizing			Externalizing		
**CBCL Threshold, Child Sex**	***Pre***	***Mid***	***Diff***	***Pre***	***Mid***	***Diff***
Borderline, Male	8.84	6.85	-1.99	11.58	7.71	-3.87
Borderline, Female	9.46	11.03	1.58	11.67	9.64	-2.02
Clinical, Male	12.71	11.30	-1.41	19.17	14.47	-4.69
Clinical, Female	16.01	12.03	-3.98	16.55	10.69	-5.86
Normal, Male	3.19	3.48	0.29	4.58	4.13	-0.46
Normal, Female	3.53	4.03	0.50	3.83	3.69	-0.14

Abbreviations: CBCL=Child Behavior Checklist; diff=difference; LS=least squares; mid=mid-pandemic; pre=pre-pandemic; ref=reference category.
Model was adjusted for time between pandemic start and survey administration, child age, child race, child ethnicity, poverty level, caregiver educational attainment, and caregiver depression.
See eTable 10 for subgroup sample sizes.

© 2024 Blackwell CK et al. *JAMA Network Open.*

eTable 15. Sensitivity analysis using continuous age variable in the generalized linear mixed-effects model estimating change in child mental health (n=1229)

	Internalizing	Externalizing	Depression	Anxiety	ADHD
**Parameter**	**β (95% CI)**	**β (95% CI)**	**β (95% CI)**	**β (95% CI)**	**β (95% CI)**
Intercept	3.81 (1.54, 6.08)	6.93 (4.28, 9.57)	3.01 (1.99, 4.04)	0.43 (-0.46, 1.32)	4.07 (2.68, 5.46)
Visit (during pandemic)	0.09 (-0.19, 0.37)	-0.88 (-1.16, -0.61)	-0.18 (-0.31, -0.05)	0.22 (0.10, 0.35)	-0.36 (-0.50, -0.22)
Time between pandemic start and survey administration	0.01 (-0.03, 0.05)	0.01 (-0.04, 0.06)	0.01 (-0.01, 0.03)	0.01 (-0.01, 0.02)	0.01 (-0.01, 0.04)
Poverty level (%FPL)					
130%	0.81 (-0.19, 1.80)	0.85 (-0.29, 1.99)	0.20 (-0.25, 0.66)	0.35 (-0.03, 0.73)	-0.05 (-0.66, 0.55)
130%-350%	0.57 (-0.12, 1.26)	0.24 (-0.58, 1.06)	0.11 (-0.24, 0.46)	0.21 (-0.06, 0.49)	0.07 (-0.35, 0.48)
>350% (ref)					
Child age, y	0.12 (-0.08, 0.32)	-0.06 (-0.29, 0.17)	-0.06 (-0.15, 0.03)	0.08 (0.01, 0.16)	-0.05 (-0.17, 0.08)
Child sex					
Female	0.23 (-0.28, 0.74)	-1.26 (-1.83, -0.69)	0.05 (-0.19, 0.29)	0.01 (-0.18, 0.20)	-1.02 (-1.31, -0.72)
Male (ref)					
Child race					
Black	-1.00 (-1.75, -0.25)	-0.14 (-0.98, 0.71)	-0.65 (-0.99, -0.30)	-0.32 (-0.60, -0.05)	-0.13 (-0.56, 0.31)
Other race	-0.28 (-1.02, 0.47)	-0.50 (-1.33, 0.34)	-0.20 (-0.55, 0.14)	-0.14 (-0.42, 0.14)	-0.44 (-0.869, -0.01)
White (ref)					
Child ethnicity					
Hispanic	-0.61 (-1.55, 0.33)	-0.82 (-1.88, 0.24)	-0.15 (-0.59, 0.29)	-0.07 (-0.42, 0.29)	-0.24 (-0.78, 0.31)
Non-Hispanic (ref)					
Caregiver educational attainment					
< High school	-0.79 (-3.01, 1.43)	0.95 (-1.53, 3.43)	-0.06 (-1.10, 0.98)	-0.04 (-0.87, 0.79)	1.43 (0.12, 2.75)
High school degree or equivalent	-0.20 (-1.44, 1.04)	1.67 (0.31, 3.04)	-0.12 (-0.64, 0.46)	0.07 (-0.39, 0.54)	0.91 (0.18, 1.64)
Some college, associate degree, or trade school	-0.30 (-1.15, 0.54)	0.53 (-0.42, 1.47)	-0.15 (-0.54, 0.25)	-0.15 (-0.46, 0.17)	0.81 (0.33, 1.30)
Bachelor's degree	-0.05 (-0.72, 0.63)	0.52 (-0.24, 1.27)	-0.09 (-0.40, 0.22)	-0.01 (-0.26, 0.24)	0.68 (0.28, 1.07)
Master's, PhD, or professional degree (ref)					
Caregiver Depression	0.12 (0.08, 0.16)	0.14 (0.09, 0.18)	0.05 (0.03, 0.07)	0.04 (0.02, 0.05)	0.05 (0.03, 0.07)
Caregiver Perceived Stress	0.10 (0.06, 0.13)	0.09 (0.05, 0.13)	0.04 (0.02, 0.05)	0.03 (0.02, 0.04)	0.05 (0.03, 0.07)

Abbreviations: ADHD = attention deficit/hyperactivity disorder; FPL=federal poverty level; ref=reference category.

© 2024 Blackwell CK et al. *JAMA Network Open.*

eTable 16. Sensitivity analysis including time between pre- and mid-pandemic assessments variable in the generalized linear mixed-effects model estimating change in child mental health (n=1229)

	Internalizing	Externalizing	Depression	Anxiety	ADHD
**Parameter**	**β (95% CI)**	**β (95% CI)**	**β (95% CI)**	**β (95% CI)**	**β (95% CI)**
Intercept	5.84 (4.27, 7.41)	5.6 (3.84, 7.35)	1.77 (1.14, 2.39)	2.07 (1.36, 2.79)	3.07 (2.18, 3.97)
Visit (during pandemic)	0.09 (-0.19, 0.37)	-0.88 (-1.16, -0.61)	0.22 (0.10, 0.35)	-0.18 (-0.31, -0.05)	-0.36 (-0.50, -0.22)
Time between pre- and mid-pandemic survey administration	-0.01 (-0.04, 0.02)	0.01 (-0.02, 0.04)	-0.01 (-0.02, 0.01)	0.003 (-0.01, 0.02)	0.01 (-0.002, 0.03)
Poverty level (%FPL)					
130%	0.83 (-0.16, 1.83)	0.86 (-0.28, 2.01)	0.37 (-0.02, 0.75)	0.22 (-0.23, 0.68)	-0.04 (-0.65, 0.56)
130%-350%	0.59 (-0.11, 1.28)	0.23 (-0.58, 1.05)	0.23 (-0.04, 0.51)	0.11 (-0.23, 0.46)	0.06 (-0.36, 0.47)
>350% (ref)					
Child age, y					
< 12	-0.22 (-1.06, 0.63)	0.44 (-0.51, 1.38)	-0.21 (-0.53, 0.12)	0.38 (-0.01, 0.77)	0.26 (-0.23, 0.75)
≥ 12 (ref)					
Child sex					
Female	0.23 (-0.28, 0.74)	-1.25 (-1.83, -0.68)	0.01 (-0.18, 0.20)	0.06 (-0.18, 0.29)	-1.01 (-1.31, -0.72)
Male (ref)					
Child race					
Black	-0.97 (-1.72, -0.22)	-0.13 (-0.97, 0.71)	-0.31 (-0.59, -0.03)	-0.64 (-0.98, -0.29)	-0.13 (-0.56, 0.31)
Other race	-0.25 (-0.99, 0.49)	-0.49 (-1.32, 0.34)	-0.13 (-0.41, 0.15)	-0.20 (-0.54, 0.15)	-0.43 (-0.86, 0.003)
White (ref)					
Child ethnicity					
Hispanic	-0.61 (-1.56, 0.33)	-0.81 (-1.87, 0.25)	-0.07 (-0.42, 0.29)	-0.15 (-0.59, 0.30)	-0.22 (-0.76, 0.33)
Non-Hispanic (ref)					
Caregiver educational attainment					
< High school	-0.73 (-2.95, 1.49)	0.97 (-1.51, 3.45)	-0.01 (-0.84, 0.82)	-0.02 (-1.06, 1.02)	1.44 (0.11, 2.76)
High school degree or equivalent	-0.16 (-1.39, 1.08)	1.70 (0.34, 3.07)	0.09 (-0.38, 0.55)	-0.08 (-0.65, 0.49)	0.93 (0.19, 1.66)
Some college, associate degree, or trade school	-0.28 (-1.12, 0.57)	0.52 (-0.42, 1.47)	-0.14 (-0.45, 0.18)	-0.15 (-0.54, 0.24)	0.81 (0.33, 1.30)
Bachelor's degree	-0.02 (-0.70, 0.65)	0.51 (-0.24, 1.27)	0.01 (-0.24, 0.25)	-0.09 (-0.40, 0.22)	0.67 (0.28, 1.07)
Master's, PhD, or professional degree (ref)					
Caregiver Depression	0.12 (0.08, 0.16)	0.14 (0.09, 0.18)	0.04 (0.02, 0.05)	0.05 (0.03, 0.07)	0.05 (0.03, 0.07)
Caregiver Perceived Stress	0.09 (0.06, 0.13)	0.10 (0.06, 0.14)	0.03 (0.02, 0.04)	0.04 (0.02, 0.05)	0.05 (0.03, 0.07)

Abbreviations: ADHD = attention deficit/hyperactivity disorder; FPL=federal poverty level; ref=reference category.

© 2024 Blackwell CK et al. *JAMA Network Open.*

eReferences

1. LeWinn KZ, Karr CJ, Hazlehurst M, Carroll K, Loftus C, Nguyen R, Barrett E, Swan SH, Szpiro AA, Paquette A, Moore P. Cohort profile: the ECHO prenatal and early childhood pathways to health consortium (ECHO-PATHWAYS). BMJ open. 2022 Oct 1;12(10):e064288.
2. LeWinn KZ, Bush NR, Batra A, Tylavsky F, Rehkopf D. Identification of modifiable social and behavioral factors associated with childhood cognitive performance. JAMA pediatrics. 2020 Nov 1;174(11):1063-72.
3. Sontag-Padilla L, Burns RM, Shih RA, Griffin BA, Martin LT, Chandra A, Tylavsky F. The urban child institute CANDLE study. Santa Monica, CA: RAND Corporation. 2015.
4. Barrett ES, Sathyanarayana S, Janssen S, Redmon JB, Nguyen RH, Kobrosly R, Swan SH, TIDES Study Team. Environmental health attitudes and behaviors: findings from a large pregnancy cohort study. European Journal of Obstetrics & Gynecology and Reproductive Biology. 2014 May 1;176:119-25.
5. Leve, L. D., Neiderhiser, J. M., Ganiban, J. M., Natsuaki, M. N., Shaw, D. S., & Reiss, D. (2019). The early growth and development study: A dual-family adoption study from birth through adolescence. Twin Research and Human Genetics, 22(6), 716-727.
6. Bruchhage MM, Ngo GC, Schneider N, D'Sa V, Deoni SC. Functional connectivity correlates of infant and early childhood cognitive development. Brain Structure and Function. 2020 Mar;225(2):669-81.
7. Wang Y, Perera F, Guo J, Riley KW, Durham T, Ross Z, Ananth CV, Baccarelli A, Wang S, Herbstman JB. A methodological pipeline to generate an epigenetic marker of prenatal exposure to air pollution indicators. Epigenetics. 2022 Jan 2;17(1):32-40.
8. Martin EM, Fry RC. Environmental influences on the epigenome: exposure-associated DNA methylation in human populations. Annual review of public health. 2018 Apr 1;39:309-33.
9. Global alliance to prevent prematurity and stillbirth. Available: https://www.gapps.org
10. Brunst KJ, Wright RO, DiGioia K, Enlow MB, Fernandez H, Wright RJ, Kannan S. Racial/ethnic and sociodemographic factors associated with micronutrient intakes and inadequacies among pregnant women in an urban US population. Public health nutrition. 2014 Sep;17(9):1960-70.

© 2024 Blackwell CK et al. *JAMA Network Open.*
