# Supplementary material for: Longitudinal Changes in Youth Mental Health From Before to During the COVID-19 Pandemic
Source: JAMA Netw Open. Author manuscript; Available in PMC 2025 Feb 25. (PMC11856357; doi:10.1001/jamanetworkopen.2024.30198)
Supplement: data sharing statement — SUPPLEMENT 3. Data Sharing Statement [file NIHMS2055771-supplement-data_sharing_statement.pdf]

Data Sharing Statement

Blackwell. Longitudinal Changes in Youth Mental Health From Before to During the COVID-19 Pandemic. *JAMA Netw Open*. Published August 26, 2024.
doi:10.1001/jamanetworkopen.2024.30198

Data
Data available: Yes
Data types: Deidentified participant data
How to access data: Select de-identified data from the ECHO Program are available through NICHD's Data and Specimen Hub (DASH). Information on study data not available on DASH, such as some Indigenous datasets, can be found on the ECHO study DASH website.
When available: beginning date: 02-01-2024

Supporting Documents
Document types: None

Additional Information
Who can access the data: Procedures to replicate these analyses are available upon request. Aside from the proprietary CBCL instrument, all measures are freely available to download from the ECHO website (https://echochildren.org/). This study was part of the ongoing ECHO research program and not preregistered.
Types of analyses: Data are available on DASH for research purposes.
Mechanisms of data availability: Data are available with permission from DASH.
Any additional restrictions: Select de-identified data from the ECHO Program are available through NICHD's Data and Specimen Hub (DASH). Information on study data not available on DASH, such as some Indigenous datasets, can be found on the ECHO study DASH website.
