# Supplementary material for: Longitudinal Changes in Youth Mental Health From Before to During the COVID-19 Pandemic
Source: JAMA Netw Open. Author manuscript; Available in PMC 2025 Feb 25. (PMC11856357; doi:10.1001/jamanetworkopen.2024.30198)
Supplement: collaborators — SUPPLEMENT 2. Environmental influences on Child Health Outcomes Program Collaborators [file NIHMS2055771-supplement-collaborators.pdf]

Supplemental Online Content: Nonauthor Collaborators
*First name, last name, and suffix (if applicable) are required and will appear in PubMed.

*Group Name(s): Environmental influences on Child Health Outcomes Cohort Consortium							
***First Name and Middle Initial(s)**	***Last Name**	***Suffix (eg, Jr, III)**	Academic Degrees	Institution	Location (city, state/province, country)	Role or Contribution, eg, chair, principal investigator	Group (if more than 1 Group listed in the byline) and/or Subgroup (eg, Steering Committee)
P Brian	Smith		MD, MPH, MHS	Duke Clinical Research Institute, Duke University School of Medicine	Durham, North Carolina, USA	ECHO Coordinating Center Principal Investigator	
L Kristin	Newby		MD, MHS	Duke Clinical Research Institute, Duke University School of Medicine	Durham, North Carolina, USA	ECHO Coordinating Center Principal Investigator	
Lisa P.	Jacobson		ScD	Johns Hopkins University, Bloomberg School of Public Health	Baltimore, Maryland, USA	ECHO Data Analysis Center Principal Investigator	
Diane	Catellier		DrPH	Research Triangle Institute	Research Triangle Park, North Carolina, USA	ECHO Data Analysis Center Principal Investigator	
David	Cella		PhD	Feinberg School of Medicine, Northwestern University	Chicago, Illinois, USA	Person-Reported OUtcome Core Principal Investigator	
Richard	Gershon		PhD	Feinberg School of Medicine, Northwestern University	Chicago, Illinois, USA	Person-Reported OUtcome Core Principal Investigator	
Julie B.	Herbstman		PhD	Columbia University Mailman School of Public Health	New York, New York, USA	ECHO Cohort Study Site Principal Investigator	
Catherine J.	Karr		MD, MS, PhD	University of Washington	Seattle, Washington, USA	ECHO Cohort Study Site Principal Investigator	
Jenae M.	Neiderhiser		PhD	Penn State University	University Park, Pennsylvania, USA	ECHO Cohort Study Site Principal Investigator	

Supplemental Online Content: Nonauthor Collaborators
*First name, last name, and suffix (if applicable) are required and will appear in PubMed.

***First Name and Middle Initial(s)**	***Last Name**	***Suffix (eg, Jr, III)**	Academic Degrees	Institution	Location (city, state/province, country)	Role or Contribution, eg, chair, principal investigator	Group (if more than 1 Group listed in the byline) and/or Subgroup (eg, Steering Committee)
Johnnye L.	Lewis		PhD	College of Pharmacy, University of New Mexico Health Sciences Center	Albuquerque, New Mexico, USA	ECHO Cohort Study Site Principal Investigator	
Debra M.	MacKenzie		PhD	College of Pharmacy, University of New Mexico Health Sciences Center	Albuquerque, New Mexico, USA	ECHO Cohort Study Site Principal Investigator	
T. Michael	O'Shea	Jr	MD, MPH	University of North Carolina School of Medicine	Chapel Hill, North Carolina, USA	ECHO Cohort Study Site Principal Investigator	
Anne L.	Dunlop		MD, MPH	Emory University School of Medicine	Atlanta, Georgia, USA	ECHO Cohort Study Site Principal Investigator	
